# Molecular Simulations with a Pretrained Neural Network and Universal Pairwise Force Fields

**DOI:** 10.1021/jacs.5c09558

**Published:** 2025-08-31

**Authors:** Adil Kabylda, J. Thorben Frank, Sergio Suárez-Dou, Almaz Khabibrakhmanov, Leonardo Medrano Sandonas, Oliver T. Unke, Stefan Chmiela, Klaus-Robert Müller, Alexandre Tkatchenko

**Affiliations:** † Department of Physics and Materials Science, 81872 University of Luxembourg, L-1511 Luxembourg City, Luxembourg; ‡ Machine Learning Group, 26524 Technische Universität Berlin, 10587 Berlin, Germany; § Berlin Institute for the Foundations of Learning and Data − BIFOLD, 10587 Berlin, Germany; ∥ Institute for Materials Science and Max Bergmann Center of Biomaterials, 9169 TUD Dresden University of Technology, 01069 Dresden, Germany; ⊥ Google DeepMind, 10117 Berlin, Germany; # Max Planck Institute for Informatics, Stuhlsatzenhausweg, 66123 Saarbrücken, Germany; ¶ Department of Artificial Intelligence, Korea University, Anam-dong, Seongbuk-gu, 02841 Seoul, Korea

## Abstract

Machine Learning Force Fields (MLFFs) promise to enable general molecular simulations that can simultaneously achieve efficiency, accuracy, transferability, and scalability for diverse molecules, materials, and hybrid interfaces. A key step toward this goal has been made with the GEMS approach to biomolecular dynamics [Unke et al., Sci. Adv. **2024**, *10*, eadn4397]. This work introduces the SO3LR method that integrates the fast and stable SO3krates neural network for semilocal interactions with universal pairwise force fields designed for short-range repulsion, long-range electrostatics, and dispersion interactions. SO3LR is trained on a diverse set of 4 million neutral and charged molecular complexes computed at the PBE0+MBD level of quantum mechanics, ensuring broad coverage of covalent and noncovalent interactions. Our approach is characterized by computational and data efficiency, scalability to 200 thousand atoms on a single GPU, and reasonable to high accuracy across the chemical space of organic (bio)­molecules. SO3LR is applied to study units of four major biomolecule types, polypeptide folding, and nanosecond dynamics of larger systems such as a protein, a glycoprotein, and a lipid bilayer, all in explicit solvent. Finally, we discuss future challenges toward truly general molecular simulations by combining MLFFs with traditional atomistic models.

## Graphical abstract

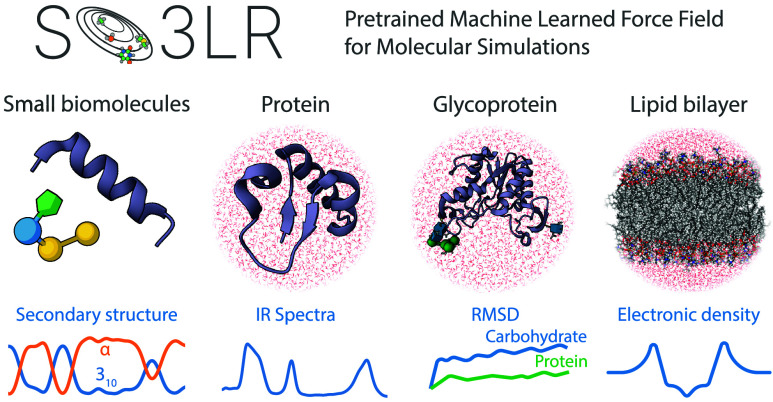

## Introduction

The desire to perform quantitative molecular dynamics (MD) simulations based solely on nuclear charges and electron numbers has been expressed by many researchers, including Schrödinger,^1^ Dirac,^2^ and Feynman.^3^ Despite a century filled with groundbreaking advances, this vision has yet to be fully realized in the realm of molecular simulations. Existing approaches often make significant trade-offs concerning **E**fficiency, **A**ccuracy, **S**calability, or **T**ransferability (EAST).^4^ In this manuscript, we argue that several methodological advances in the field of atomistic modeling have coalesced to bring us closer to achieving fully quantitative, quantum-accurate molecular simulations. While the journey toward this ultimate goal may be lengthy and complex, it is a pursuit that is undeniably worthwhile and requires a collaborative community-based effort.

A key challenge in molecular simulations is the construction of an atomistic force field model that satisfies the EAST requirements mentioned above.^5–12^ Traditionally, force fields are obtained either from approximate but fast mechanistic expressions, or accurate but computationally prohibitive *ab initio* electronic-structure calculations. Both approaches compromise either accuracy or efficiency, restricting the scope of problems that can be addressed. Recently, machine-learned force fields (MLFFs) have started to bridge this gap by exploiting statistical models with high flexibility.^10–14^ Unlike classical force fields, MLFFs exhibit unprecedented transferability across chemical space; however, scalability with system size remains an issue.

Many challenges remain to be addressed to enable EAST-compliant and MLFF-driven general molecular simulations. Among these we mention the development of data and computationally efficient semilocal interatomic interaction models,^15–24^ explicit treatment of (many-body) long-range interactions,^12,25,26^ building data sets with comprehensive coverage of chemical space,^27–35^ and development of modern GPU-enabled molecular simulation frameworks.^36–38^ Within this work, we take decisive steps toward solving the aforementioned challenges for organic (bio)­molecules. Our solution combines recent advances from chemical and computational physics, machine learning (ML), and established techniques from the force field community. Semilocal interactions are described by the SO3krates ML model^39^ using a many-body anharmonic treatment. The physical pairwise terms include short-range Ziegler-Biersack-Littmark repulsion,^40^ long-range electrostatic interactions, and a recently derived universal interatomic van der Waals (vdW) dispersion potential.^41^ Complementarity between the different terms is achieved through careful parametrization on a curated and comprehensive data set of 4M molecular structures computed with essentially nonempirical and widely applicable PBE0+MBD functional, leading to the SO3LR model (we suggest pronunciation “solar”).

We demonstrate the applicability and stability of SO3LR in nanosecond-long simulations of small biomolecular units, polyalanine systems, bulk water, crambin protein, N-linked glycoprotein, and a lipid bilayer. SO3LR can be scaled to simulations involving up to ∼200k atoms with a latency of ∼3 μs/atom/step on a single H100 GPU, thus approaching sizes and time scales relevant for realistic biomolecules.

## Results

### SO3LR Components

Generally applicable molecular simulations can be directly related to an accurate description of interactions across systems and length scales. To achieve these objectives, SO3LR decomposes the potential energy into four contributions (Figure 1A):

$$E_{\mathrm{Pot}}=\;E_{\mathrm{ZBL}}+E_{\mathrm{SO}3k}+E_{\mathrm{Elec}}+E_{\mathrm{Disp}}$$ 1

where *E*_ZBL_ is a short-ranged term inspired by Ziegler–Biersack–Littmark (ZBL) repulsion between nuclei (see Supporting Information for more details), *E*_SO3k_ is the semilocal many-body potential learned by the SO3krates model, and *E*_Elec_ and *E*_Disp_ are the long-ranged electrostatic and dispersion energies, respectively. All potential terms influence each other, and a careful optimization procedure based on a diverse data set of ∼4 million points ensures a broad applicability. The proposed combination of model design, data set curation and joint optimization, resolves the trade-offs in the EAST requirements and is described in the following paragraphs.

**Figure 1:**
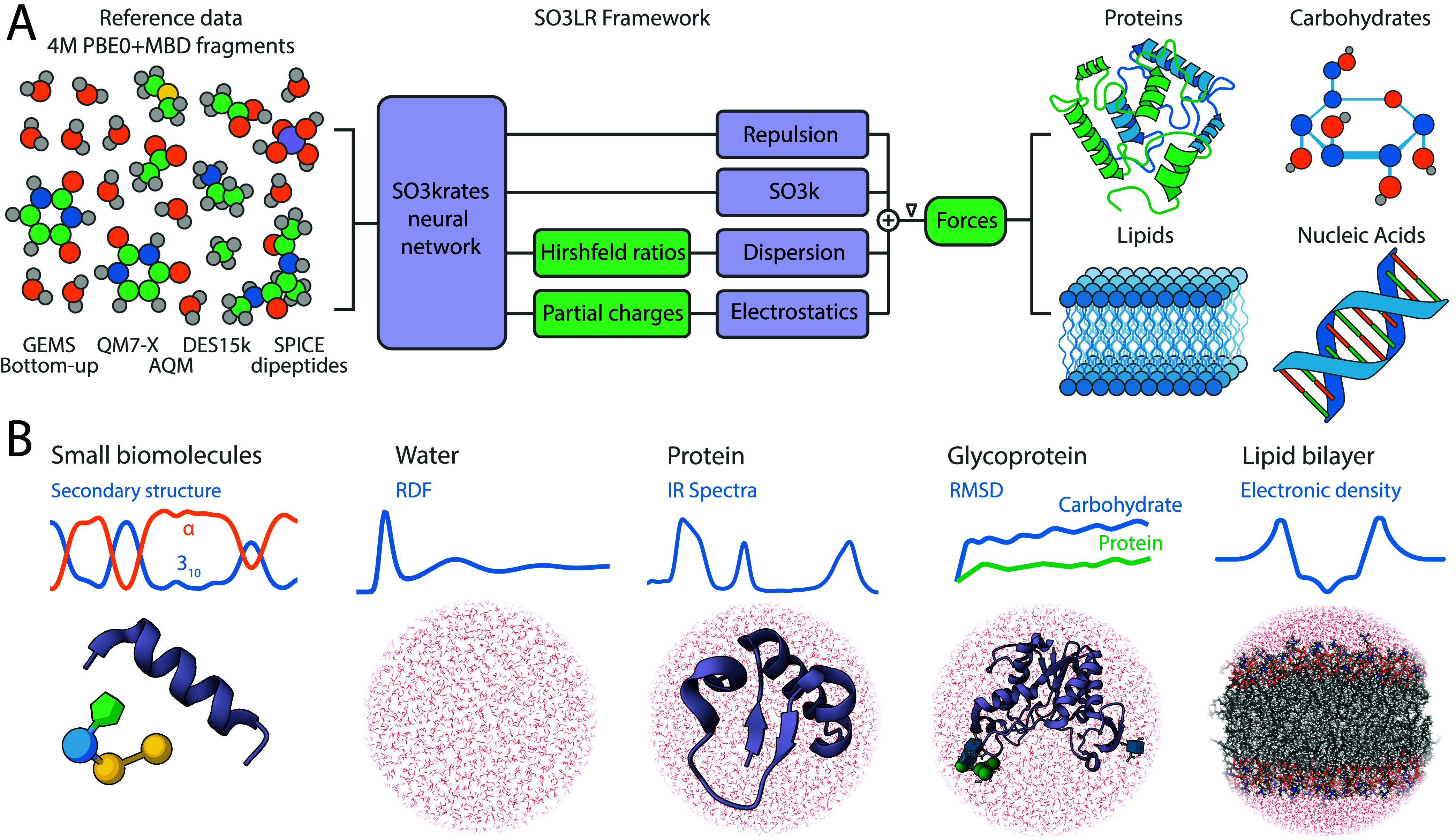
Overview of the SO3LR model and simulation results. (A) SO3LR combines the SO3krates neural network with physically inspired interactions, including ZBL repulsion, electrostatics, and a universal pairwise van der Waals potential for dispersion, which interact directly with the neural network model. All building blocks are jointly trained on a carefully curated data set which covers a broad range of chemical space and interaction classes. SO3LR enables simulations of small biomolecular units of all four major types of biomolecules, and large-scale simulations of three types. (B) This includes large-scale simulations of liquid water, protein, glycoprotein, and a lipid bilayer.

#### EA – SO3krates

The cornerstone of our approach, which enables high computational efficiency and accuracy (**EA**ST), is the SO3krates model^22,39^ – an MLFF based on an equivariant graph neural network (a compact introduction to invariance and equivariance is given in the Supporting Information). Starting from atomic positions *R*, atomic numbers *Z*, total charge *Q*, and total spin *S*, it predicts atomic quantities:

$$E_{i},q_{i},h_{i}=\mathrm{SO}3\mathrm{krates}\left(R,Z,Q,S\right)$$ 2

where *E*_*i*_ are atomic energies, *q*_*i*_ are partial charges and *h*_*i*_ are Hirshfeld ratios (ratio of effective and free-atom volume, *V*_eff_/*V*_free_).^42^ The semilocal energy contribution is then calculated as the sum over the atomic energies:

3 $$E_{\mathrm{SO}3k}=\overset{N}{\underset{i=1}{\sum }}E_{i}$$

The predicted atomic energies contain information about atoms in the direct local neighborhood *and* beyond via mean field updates, which is why we refer to the energy prediction as semilocal. The mean-field nature of these updates cannot account for all types of interactions and is limited by an effective cutoff, which is *upper bounded* by the local cutoff times the number of update steps (the effective cutoff in SO3LR is 13.5 Å).

#### S – Long-Range Dispersion and Electrostatics

To improve the description of long-range effects and extend the description beyond semilocal environments, we explicitly incorporate electrostatics and universal pairwise interatomic vdW potentials. Both partial charges and vdW parameters depend on the atomic environment and are predicted by the SO3krates neural network. As shown in Figure S1, the distributions of Hirshfeld ratios and partial charges for AcAla_15_NMe exhibit substantial element- and environment-specific variability. For example, Hirshfeld ratios for hydrogen span a broad range from 0.55 to 0.8, partial charges of carbon and nitrogen range from −0.4 to −0.3. The contributions of these long-range interactions to atomic forces are computed using automatic differentiation tools; hence, the variation of charges and vdW parameters with atomic displacements is fully accounted for. Both long-range terms follow the correct pairwise asymptotic decay. This is an important requirement for the scalability (EA**S**T) to length scales that exceed those covered by the training data.

*Dispersion* interactions are calculated using universal pairwise interatomic vdW potentials derived from quantum Drude oscillators (QDO):^41^

$$E_{\mathrm{Disp}}=-\underset{i<j}{\sum }\overset{5}{\underset{n=3}{\sum }}\frac{C_{2n}^{ij}}{r_{ij}^{2n}+R_{d,\mathrm{ij}}^{2n}}$$ 4

where *C*_2*n*_^*ij*^ are long-range interatomic dispersion coefficients, and *R*_*d*,*ij*_^2*n*^ are the vdW radii of the Becke-Johnson damping function.^43^ The radii are defined based on atomic polarizabilities as:^44^

$$R_{d,\mathrm{ij}}=\gamma R_{\mathrm{vdW}}^{ij}=2\gamma {\left(\frac{a_{0}^{4}\alpha_{\mathrm{fsc}}^{-4/3}}{4\pi \varepsilon_{0}}\frac{\alpha_{i}+\alpha_{j}}{2}\right)}^{1/7}$$ 5

where *a*_0_ is the Bohr radius, α_fsc_ = *e* ^2^/4*πε*_0_ℏ*c* is the fine-structure constant, with γ being a single tunable parameter in the dispersion module that controls the damping strength. Atomic polarizabilities α_*i*_ and dipole–dipole dispersion coefficients *C*_6_^*ij*^ are obtained using the Tkatchenko-Scheffler method^45^ with the ML-predicted Hirshfeld ratios *h*_*i*_, whereas the scaling relations from the QDO model^41,46^ are applied to generate higher-order dispersion coefficients *C*_8_^*ij*^ and *C*_10_^*ij*^.

*Electrostatic* interactions are modeled using a damped Coulomb potential:

6 $$E_{\mathrm{Elec}}=\underset{i<j}{\sum }q_{i}q_{j}\frac{\mathrm{erf}\left(r_{ij}/\sigma \right)}{r_{ij}}$$

where *q*_*i*_ are the ML-predicted partial charges, and σ is a hyperparameter that controls the damping strength. We remark that the semilocal SO3k module has the capacity to accurately describe multipolar interactions; hence, we limit our model to leading-order electrostatics.

*Coupling between long-range and semilocal* energy contributions arises from the structure of the potential energy prediction (eq 1). The long-range modules have a nonzero energy contribution for all atomic pairs, including those within the local cutoff of the MLFF. As such, the functional forms of the long-range potentials alter the potential which is learned by the SO3krates model. The choice of damping hyperparameters γ and σ controls the fine balance between semilocal, electrostatic and dispersion interactions. In principle, SO3krates can learn to correct for arbitrary choices of σ and γ up to the local cutoff; however, damping particularly impacts dynamical behavior in MD simulations, although the overall model performance remains largely unaffected. This can be attributed to the fact that semilocal and long-range interactions are coupled nonlinearly through parameter and hyperparameter optimization in both modules. Hence, the damping hyperparameters were fine-tuned on the S66x8 benchmark data set.^47^

#### T – Optimization on Diverse Training Data

All SO3LR modules are jointly optimized on a diverse data set that spans a broad chemical space and various interaction classes. This enables transferability (EAS**T**) between all four major types of biomolecules.

*The comprehensive data set* has been a key factor in the development of our MLFF. It is a collection of extensive quantum mechanical data from both small and large molecules, as well as noncovalent systems with and without explicit solvation. To this end, we combined five data sets: 2.7M bottom-up GEMS fragments,^48^ 1M QM7-X molecules,^32^ 60k AQM gas-phase molecules,^35^ 33k SPICE dipeptides,^34^ and 15k DES molecular dimers^31^ (see Figure S2 and Table S1 for more details). The first three data sets were originally computed at the PBE0+MBD level of theory.^49,50^ For consistency, we recomputed the remaining two data sets using the same reference method.

The PBE0+MBD method combines the nonempirical hybrid functional with an explicit treatment of many-body long-range dispersion interactions. This level of theory has been shown to yield excellent agreement with both high-level quantum chemistry methods and experimental data. Its accuracy has been demonstrated for a wide range of systems, including polypeptides,^51,52^ supramolecular complexes,^53^ and molecular crystals.^54,55^

The data sets are complementary in terms of conformational space and chemical diversity, covering 8 elements predominantly present in biosystems (H, C, N, O, F, P, S, and Cl). Specifically, the QM7-X data set encompasses the chemical space of small organic molecules, while the AQM data set includes medium-sized drug-like molecules. DES molecular dimers were incorporated to improve the description of noncovalent interactions. SPICE dipeptide structures were added to enhance the accuracy for the protein-containing systems. Lastly, the GEMS bottom-up data set contains gas-phase and explicitly microsolvated protein fragments, as well as structures with gas-phase water clusters.

A natural and fundamental question concerns whether the 4M molecular conformations used to train SO3LR adequately span the chemical space relevant to (bio)­molecular systems. Although a comprehensive assessment of this coverage remains an open challenge for future investigation, approximate estimates offer valuable insight. For example, chemically accurate simulations of medium-sized peptides, such as alanine tetrapeptide, have been demonstrated using fewer than 1,000 conformations when using SO3krates as the underlying MLFF.^39^ Extrapolating this to the entire combinatorial space of tetrapeptides composed of the 20 natural amino acids yields a naïve estimate of approximately 160M conformations required for complete coverage. This is a significant overestimate, primarily because of the high redundancy of local chemical environments,^56^ a property that underpins the transferability of foundational MLFF models across families of molecules or materials.

Indeed, a meaningful measure of chemical diversity can be captured by the number of *orbits* – distinct equivalence classes of atoms that possess identical local environments across different molecular configurations.^57^ The number of orbits depends on the effective distance cutoff used to build molecular subgraphs. For the alanine tetrapeptide, there are 10 orbits up to second neighbors, meaning that 100 conformations per orbit is a sufficient training size. We took several data sets with published geometries (the data set used to train SO3LR, SPICE,^34^ GDB-13 ^27^) and calculated the number of orbits by building graphs up to second neighbors. By doing so, we obtained a range of 10,000–50,000 orbits for molecular data sets containing 8–10 atomic species. This preliminary analysis demonstrates that between 1 and 5M molecular configurations should be enough to cover a broad chemical space of (bio)­molecular systems. This analysis, of course, holds true only because SO3LR uses message passing and an explicit physical model for long-range interactions, meaning that only shorter-range orbits need to be accurately captured by the graph neural network architecture.

*Optimization* of the model parameters is done by minimizing a combined loss:

7 $$L=\frac{\lambda_{F}}{B}\overset{B}{\underset{b=1}{\sum }}\frac{1}{N_{b}}\overset{N_{b}}{\underset{i=1}{\sum }}{\left|\left|{\mathrm{F⃗}}_{i,\mathrm{true}}-{\mathrm{F⃗}}_{i,\mathrm{pred}}\right|\right|}_{2}^{2}+\frac{\lambda_{\mu }}{B}\overset{B}{\underset{b=1}{\sum }}{\left|\left|{\mathrm{\mu ⃗}}_{b,\mathrm{true}}-{\mathrm{\mu ⃗}}_{b,\mathrm{pred}}\right|\right|}_{2}^{2}+\frac{\lambda_{h}}{B}\overset{B}{\underset{b=1}{\sum }}\frac{1}{N_{b}}\overset{N_{b}}{\underset{i=1}{\sum }}{\left(h_{i,\mathrm{true}}-h_{i,\mathrm{pred}}\right)}^{2}$$

where *F⃗*_*i*_ are atomic forces, μ⃗ are molecular dipoles, and *h*_*i*_ are Hirshfeld ratios, with λ as trade-off parameters between the individual loss terms. The Hirshfeld ratios and partial charges are predicted by SO3krates (eq 2), and the forces are obtained as the gradient *w.r.t.* the atomic positions of the potential energy (eq 1). The partial charges are indirectly trained based on dipole moments, instead of direct fitting to reference partial charges. This approach reduces the model’s sensitivity to the choice of charge-equilibration scheme and enhances transferability.^58^ It should be noted that the model is trained on forces, rather than on energies and forces, which ensures accuracy of relative energy predictions only. Further training details can be found in the Supporting Information.

### SO3LR Evaluation

A force field that is truly EAST-compliant should be able to accurately simulate systems of varying nature and size. To demonstrate the capabilities and limitations of SO3LR, we first evaluate its performance on test and benchmark sets to assess its precision in predicting forces, binding energies, dipole moments, and Hirshfeld ratios. This is followed by an analysis of the dynamics of small biomolecular units from the MD22 benchmark data set.^59^ We then investigated the folding and stability of polyalanine systems *in vacuo*, which depend on a delicate interplay of various interactions. Before transitioning to simulations of larger biosystems, we performed a detailed analysis of water dynamics. Finally, we extend the evaluation to large-scale MD simulations of more complex systems, including a protein, a glycoprotein, and a lipid bilayer, all in explicit water (see Figure 1B).

#### Test Set and Benchmark Errors

We begin the evaluation of the model by analyzing its accuracy *w.r.t.* quantum mechanical reference data (Table 1). The test set comprises 10k randomly sampled structures from each of the QM7-X and GEMS bottom-up fragments (all other training sets were fully utilized during training). Furthermore, we recalculated 100 random structures from six MD22 reference molecules at the PBE0+MBD/tight level of theory, and evaluated the model using ∼300 AcAla_15_NMe structures and ∼5600 top-down fragments of crambin that were used in the training of system-specific models in ref 48.

**Table 1:** Root Mean Square Error of the Model on Various Test Sets [Force (eV/Å), Dipole Moment Vector (e × Å), and Hirshfeld Ratios]^a^

Data set	Size	# atoms	Force	Dipole	Hirsh. rat.
QM7-X	10000	6–23	0.069	0.031	0.012
GEMS bottom-up	10000	2–120	0.086	0.048	–
AcAla_3_NMe	100	42	0.052	0.051	0.012
DHA	100	56	0.053	0.072	0.012
AT-AT	100	60	0.168	0.238	0.025
Stachyose	100	87	0.105	0.119	0.016
Buckyball catcher	100	148	0.384	4.030	0.029
Nanotube	100	370	0.717	2.950	0.039
AcAla_15_NMe	312	162	0.055	–	–
Crambin top-down	5624	230–321	0.057	–	–
TorsionNet500	12000	13–37	0.088	0.061	0.019

a Dash indicates no data.

The model demonstrates good performance in predicting forces, dipole moments, and Hirshfeld ratios. A closer examination of the table reveals two key observations. First, fragments from curved carbon-based systems, such as the buckyball catcher and double-walled nanotube, are absent from the training set, which is reflected in the increased errors. This suggests that further expansion of the data set would be necessary to achieve complete transferability across the chemical space. Nevertheless, the MD simulations of the six MD22 molecules remain stable, as discussed in the following subsection. It is important to note that the commonly reported MD22 errors^23,24,39^ correspond to system-specific models, which evaluate the performance in distribution and are therefore lower. Second, by comparing the nearly identical errors for AcAla_3_NMe, AcAla_15_NMe, and crambin top-down fragments, we conclude that the SO3LR model is scalable to large solvated protein fragments and that long-range modules effectively describe intermolecular interactions, despite being trained only on small fragments.

To assess the model’s accuracy on conformational energetics, we evaluated torsional energy profiles using the TorsionNet500 benchmark,^62^ recomputed at the PBE0+MBD level of theory (Figure S3). The model achieves a mean absolute error (MAE) of 1.03 kcal/mol, demonstrating accurate performance across diverse torsional motifs commonly encountered in biosimulations. It should be mentioned that the absence of certain functional groups, such as triazole and trifluoromethylthio moieties, in the training set significantly increases the average errors.

To evaluate the quality of electrostatic interactions, we benchmarked partial charge prediction using the QM7b and AlphaML data sets, which were computed at the LR-CCSD/d-aug-cc-pVDZ level of theory.^60^ SO3LR accurately predicts dipole moments with an MAE of 0.13 D in magnitude and 5.1° in angle orientation (Figure 2A). This performance is comparable to hybrid DFT at the B3LYP/d-aug-cc-pVDZ level of theory, which attains 0.09 D.^60^ Our training set contains molecules from the QM7-X data set, which includes perturbed structures from QM7b. The AlphaML benchmark, on the other hand, contains a wider set of compounds, including DNA/RNA nucleobases, amino acids, carbohydrates, drugs, and hydrocarbons. Both B3LYP/d-aug-cc-pVDZ and PBE0+MBD/tight methods yield an MAE of 0.10 D on this data set, while SO3LR achieves an MAE of 0.14 D (Figure 2A), showcasing transferable and accurate prediction of dipole moments, which is crucial for calculating reliable electrostatic interactions.

**Figure 2:**
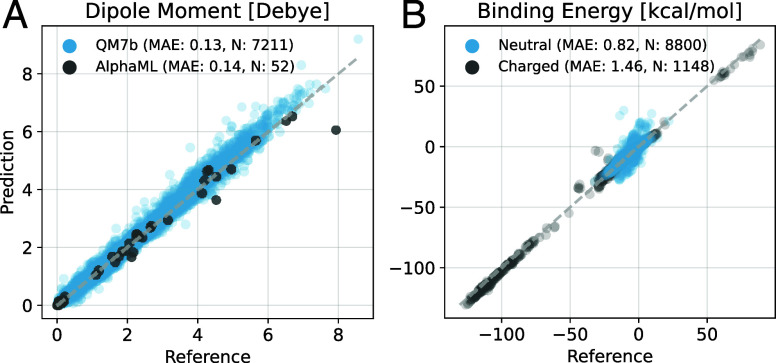
Evaluation of the SO3LR long-range modules’ performance. (A) Evaluation of the model on dipole moment prediction for 7k QM7b molecules and AlphaML showcase database.^60^ (B) Performance of the model evaluated on the unseen SAPT10k data set,^61^ separated into neutral and charged subsets.

Next, we evaluate noncovalent interaction energies on a comprehensive SAPT10k benchmark computed at the SAPT2+(3)­(CCD)/aug-cc-pVTZ level of theory.^61^ It consists of 70 subsets, featuring challenging binding motifs dominated by electrostatics and/or dispersion interactions and offering substantial diversity across chemical space. We exclude 34 out of 9982 complexes because they contain atom types beyond the 8 elements our model was trained on (hence, predictions on those structures are not meaningful). Overall, the model performs well, achieving subchemical accuracy with an MAE of 0.90 kcal/mol (Figure 2B). Rare outliers with errors up to 40 kcal/mol include complexes with exotic molecules absent from the training set, such as ClF, P­(CNO)_3_, PH_2_NO_2_. Recalculation of these outliers at the PBE0+MBD level confirms the errors arise from missing training data rather than from the reference method (Figure S4). This is a remarkable performance overall, particularly since part of the error comes from the difference between the CCD and PBE0+MBD reference levels.

#### Simulations of Small Biomolecular Units

Molecular dynamics simulations are the ultimate test for evaluating force fields. We simulated six molecular systems from the MD22 benchmark, encompassing four major biomolecule types and two supramolecular complexes: the AcAla_3_NMe tetrapeptide, stachyose tetrasaccharide, AT-AT DNA base pairs, docosahexaenoic fatty acid (DHA), the buckyball catcher, and the double-walled nanotube. The first two systems underwent 500 ps of simulation at 500 K to compare with the PBE+MBD references computed at 500 K; other systems were simulated at 300 K. The model demonstrated robust conformational exploration across all molecules. In particular, the free-energy surface exploration of tetraalanine and stachyose closely aligns with MD22 *ab initio* results, computed at the PBE+MBD level of theory, as shown in Ramachandran plots (Figure 3). Note that in this figure we report only short MD simulations to match the length of DFT simulations, and the comparison between PBE+MBD and SO3LR dynamics is only provided as a guide to the eye. Full 500 ps trajectories are shown in Figure S5.^63^ The tetrapeptide explores all ‘allowed’ (ϕ/ψ) regions found in experimental protein structures.^63^ The buckyball catcher and double-walled nanotube complexes remained stable (Figure S6), despite larger errors on the test set. This highlights the stability of SO3LR, even when applied to systems far outside its training domain. Overall, these results suggest that our model can reliably explore conformational landscapes of small molecules even in the absence of the system-specific training data.

**Figure 3:**
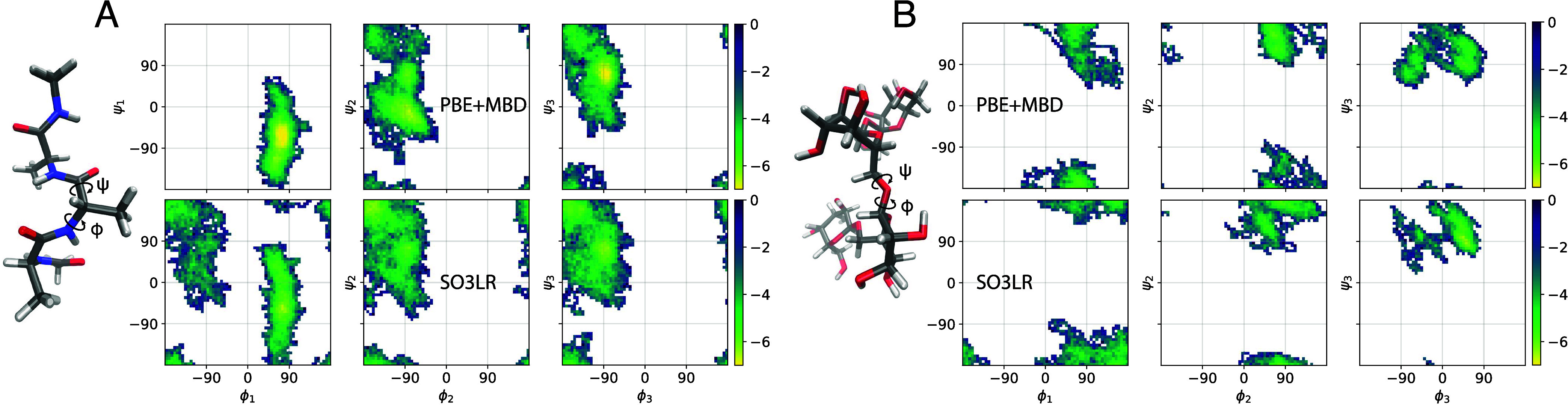
Simulations of small biomolecular fragments. Ramachandran plots (ϕ/ψ dihedrals) for (A) AcAla_3_NMe and (B) stachyose from the MD22 data set.^59^ PBE+MBD and SO3LR simulations at 500 K with 85 ps for AcAla_3_NMe and 27 ps dynamics for stachyose. SO3LR simulations of 500 ps are shown in Figure S5. Trajectory is sampled every 1 fs. The Boltzmann-inverted scale is shown in kcal/mol. The comparison between PBE+MBD from MD22 and SO3LR (trained on PBE0+MBD) is only shown as a guide to the eye.

#### Simulations of Polyalanine Systems

We further investigate polyalanines, focusing on the folding of extended AcAla_15_NMe and the stability of the folded AcAla_15_LysH^+^ at elevated temperatures. These systems present significant challenges due to the delicate interplay of hydrogen bonding, polarization, and dispersion interactions. Previous attempts to simulate them without incorporating top-down fragments either failed to correctly fold AcAla_15_NMe or overstabilized the α-helix, and in some cases, predicted diminished stability for AcAla_15_LysH^+^.^48,64^

For each system, we performed four runs of 500 ps. The extended AcAla_15_NMe structure folded in all cases (Figure 4A and Figure S7A). The time scales and folding mechanisms were similar to those observed in ref 48: initially, the peptide primarily consists of turns, then passes through a “wavy” intermediate, and finally folds into a helical form with dynamic transitions between α- and 3_10_-helices. The latter is particularly noteworthy, as empirical force fields tend to overestimate the stability of α-helices.^65,66^

**Figure 4:**
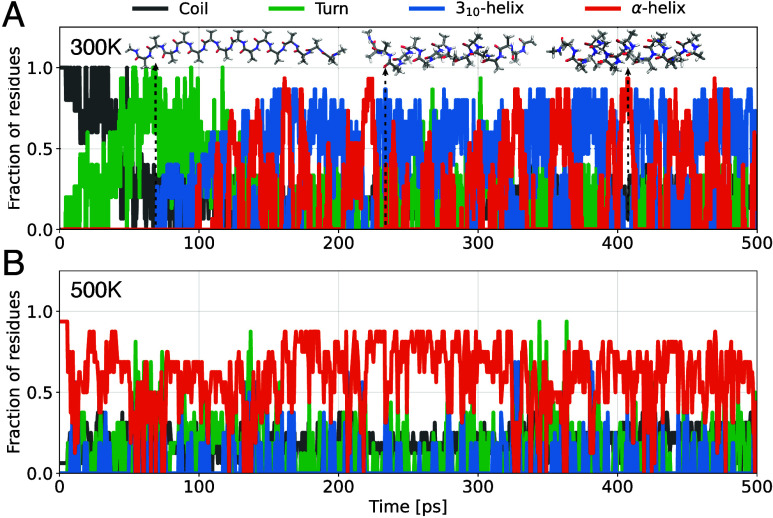
Simulations of polyalanines. (A) Secondary structural motifs observed along a typical folding trajectory of AcAla_15_NMe at 300 K in gas phase. (B) Secondary structural motifs observed along a trajectory of AcAla_15_LysH^+^ at 500 K in gas phase, starting from the folded α-helix conformation.

For the folded AcAla_15_LysH^+^, we observe that the α-helical motifs are preserved up to 500–600 K (Figure 4B and Figure S7B). These findings agree with experimental measurements, which observe scattering cross sections for AcAla_15_LysH^+^ consistent with an α-helical structure up to ≈725 K when subject to interactions with the helium buffer gas.^71^ Direct comparison with gas-phase experiments would have to explicitly include the helium environment and quantum nuclear effects. Overall, the two polyalanine systems provide a good evaluation of scalability to medium-sized systems in dynamics, complementing the observed scalability in terms of test errors.

#### Simulations of Liquid Water

Liquid water plays a crucial role in biosystems, making it an essential subject for SO3LR’s evaluation. We performed a simulation of a water box containing 4096 water molecules in the NPT ensemble. Observables were averaged over 300 ps following an initial 200 ps equilibration phase. Our analysis focused on three aspects: radial distribution function, density, and self-diffusion coefficient.

The oxygen–oxygen radial distribution function shows the expected shell structure (Figure S8), which indicates, however, that the liquid phase is slightly overstructured. Increasing the temperature to 330 K allows for an approximate treatment of missing nuclear quantum effects and improves agreement with the experimental data.^72,73^ The water density varies between 1.04 and 0.97 g/cm^3^ for long-range cutoffs of 10–20 Å (Figure S9). We adopted a cutoff of 12 Å for all subsequent biosimulations in explicit water, balancing accuracy and computational efficiency. The calculated self-diffusion coefficient is 0.079 Å^2^/ps at 300 K and 0.224 Å^2^/ps at 330 K with the 12 Å long-range cutoff. For comparison, the experimental diffusion coefficient at room temperature is 0.23 Å^2^/ps.^74^

The SO3LR results agree well with explicit *ab initio* MD using the PBE0+vdW functional.^72^ This is notable given that the training data set contains only gas-phase water clusters with at most 40 molecules (∼10k clusters or ∼0.26% of the combined data set). It is known that *ab initio* MD simulations with the PBE0+vdW functional struggle to fully capture many experimental properties of water, mainly due to the tetrahedral H-bond arrangement that amplifies the slight overestimation of PBE0 for individual hydrogen bonds.^72^ Consequently, the MLFF performance cannot and should not exceed the accuracy of the underlying *ab initio* calculations. The description of water could be improved by using higher-level *ab initio* data, such as coupled-cluster or quantum Monte Carlo methods, and by explicitly incorporating nuclear quantum effects in MD simulations. For biomolecules in water, hydrogen bonding is just one of many contributing interactions, and we have shown that accurate biomolecular dynamics can be carried out with MLFFs trained on PBE0+MBD data provided that the density of water is correctly reproduced.^48^

#### Simulations of Large Biomolecules

Finally, we showcase the potential of SO3LR by simulating large biomolecules in explicit water. The selected systems encompass various classes of biomolecular components, each characterized by distinct structural and functional properties that can be validated against existing simulations or experimental data. The systems include the crambin protein, glycoprotein (PDB: 1K7C), and the POPC lipid bilayer.

For crambin (25k atoms including water), we compute the power spectrum from 125 ps of dynamics at a temporal resolution of 2.5 fs, after a 1 ns equilibration period. The experimental water vibrations at 1640 cm^–1^ and 3200–3600 cm^–1^ are reproduced in SO3LR with better agreement than GEMS, AMOEBA and AmberFF (Figure 5A). We further examine the root mean square deviation RMSD­(t, t+Δt) averaged over three 3 ns simulations, indicating that SO3LR shows slightly increased protein mobility on longer time scales, consistent with the GEMS model (Figure 5B). We find that the overall structure stays folded during the simulation, without any indication of unfolding or bond breaking (Figure S10). To visualize conformational space sampling, we applied the two-dimensional uniform manifold approximation and projection (UMAP).^68^ The projection of the paths reveals that SO3LR and GEMS sample the conformational space more extensively than AmberFF and AMOEBA (Figure 5C), which aligns well with the high conformational variability derived from NMR measurements.^67^

**Figure 5:**
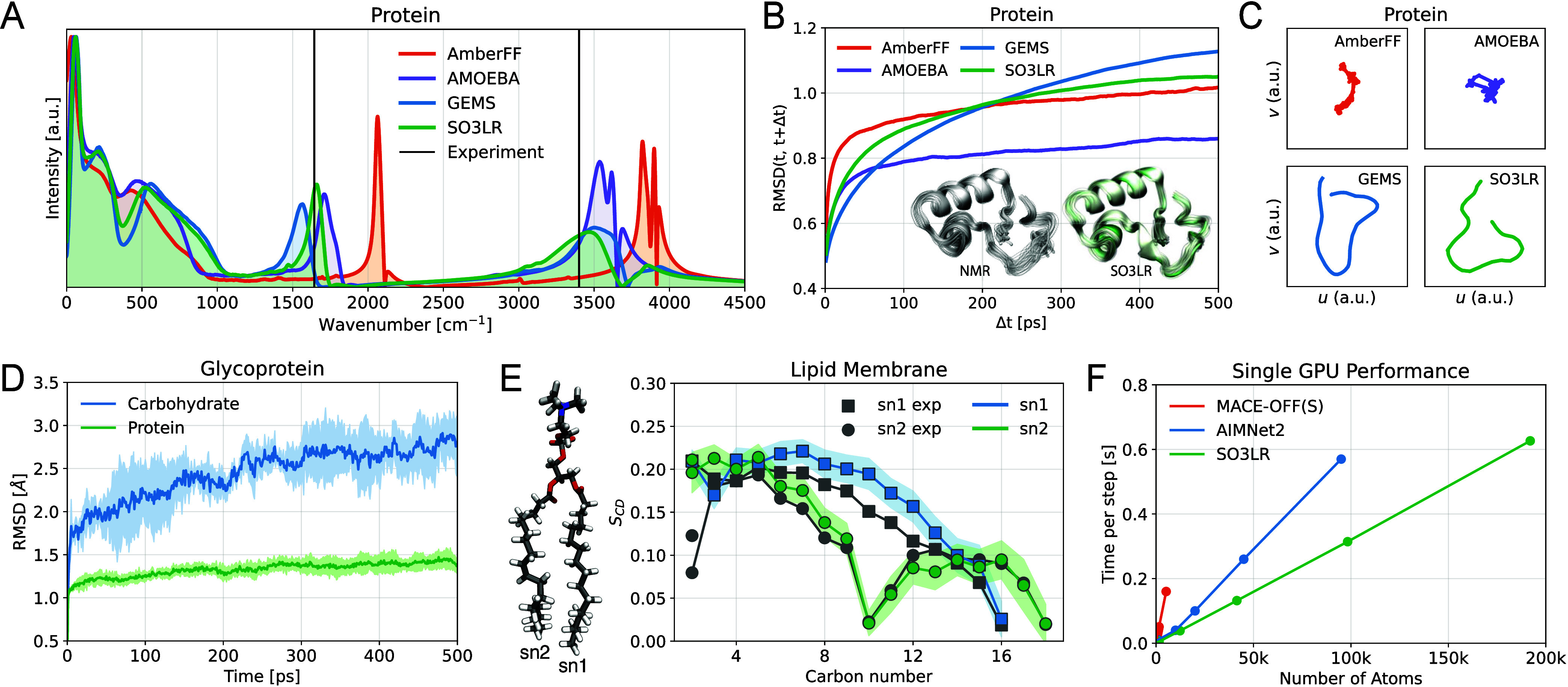
Simulations of explicitly solvated biomolecules. (A) Power spectrum of crambin in water obtained from 125 ps of dynamics. AmberFF and GEMS results are taken from ref 48. (B) Root mean square deviation (RMSD) of Crambin, excluding hydrogen atoms, between conformations sampled at times *t* and *t* + Δ*t* averaged over three 3 ns runs. The inset shows an overlay of frames from the SO3LR trajectory and NMR-derived protein structures.^67^ (C) Two-dimensional Uniform Manifold Approximation and Projection (UMAP)^68^ embedding of crambin simulation trajectories. The same latent space projection is used across all subplots. (D) RMSD of protein and carbohydrate segments of glycoprotein averaged over three 500 ps runs. (E) Tail group NMR order parameters from SO3LR simulation of the 128 POPC Lipid Bilayer and from experiment.^69^ The standard deviation is shown with background color. (F) Single GPU performance. SO3LR latencies were measured based on liquid water molecular dynamics using JAX-MD^37^ in the NVT ensemble on an H100 80 GB GPU. The slope is 3.25 × 10^–6^ s/atom/step. Latencies for MACE-OFF­(S) and AIMNet2 were measured on A100 and H100, respectively, and are taken from refs 64, 70 .

For glycoprotein (48k atoms including water), we conducted a 500 ps simulation at 300 K. This system, which comprises both protein and carbohydrate segments, presented a challenge for SO3LR due to the absence of carbohydrates in its training data. Despite this, the model successfully inferred increased carbohydrate flexibility, as evidenced by the greater RMSD observed for the carbohydrate segment compared to the protein segment (Figure 5D). These findings align with the results of the CHARMM force field specifically tuned for carbohydrates.^75^ However, the simulation revealed limitations in sampling conformations of the N-linkage. Specifically, the C_γ_–C_β_–C_α_–N dihedral, located at the protein–carbohydrate junction, can adopt three conformations: *g*+ (60°), *anti* (180°), and *g*– (300°). Our simulation only sampled the anticonformation out of these three possible states. Longer simulations would be required to determine whether the model can explore other conformations without carbohydrate–protein linkages in the training data set.

Lastly, we modeled a homogeneous POPC lipid bilayer (33k-atom system consisting of 128 lipids and 5120 water molecules). We performed a 500 ps simulation at 303 K and examined the structural properties: area per lipid, bilayer thickness, and lipid tail order parameters. These properties are critical measures of the accuracy of lipid simulations and are highly sensitive to factors such as hydrophilic attraction between head groups, hydrophobic repulsion between lipid tails, and interactions with surrounding water molecules. We found that SO3LR is in good agreement with experimental data and with empirical force fields specifically fine-tuned to lipid simulations (Table 2). The 10% underestimation of the area-per-lipid likely stems from the isotropic NPT ensemble currently used in SO3LR simulations, compared to the semi-isotropic NPT used for empirical force fields. NMR lipid tail order parameters are another important quantitative measure describing the degree of order within the acyl chains of lipids in a bilayer. The order parameters averaged over the last 250 ps suggest that the bilayer structure is in suitable agreement with NMR experiments (Figure 5E).^69^

**Table 2:** POPC Lipid Bilayer Structural Properties: Bilayer Thicknesses *D*_*HH*_ (Å) and Area Per Lipid (Å^2^)

	*D*_*HH*_	S/lipid
Experiment^76^	36.5	64.3 ± 1.3
Lipid21 ^77^	38.50 ± 0.20	63.92 ± 0.09
CHARMM36/LJ-PME^78^	37.3 ± 0.3	65.4 ± 0.5
SO3LR	37.0	58.0 ± 0.1

To assess the computational performance of SO3LR, we conducted NVT simulations of water boxes ranging from 1,536 to 192,000 atoms using JAX-MD on a single GPU (Figure 5F). The measured scaling corresponds to a slope of 3.25 × 10^–6^ s/atom/step, enabling simulations of 2.6 ns/day for a 10,000-atom system using a 1 fs time step. This performance allows nanosecond-scale simulations of large solvated systems on standard hardware. However, simulations of intricate conformational changes that occur on millisecond time scales, such as solvated protein folding, remain beyond reach at present. The current SO3LR model, with 128 features, 3 interaction layers, and a 4.5 Å local cutoff, was designed to balance accuracy and speed. These hyperparameters can be systematically adjusted to trade accuracy for performance: smaller models can be trained to accelerate simulations while maintaining chemical fidelity.^79^ Notably, the presented SO3LR model was trained on a single GPU using a modest computational budget of 86 GPU hours.

## Discussion and Conclusions

A long-held vision in the atomistic simulation community is the development of force fields with a unified functional form that can be applied across diverse chemical spaces – such as solvents, proteins, DNA, RNA, sugars, and lipids. These force fields should closely approximate quantum-mechanical behavior while remaining efficient and scalable enough to model realistic biomolecular complexes under various conditions (e.g., pressure, temperature, and external environments). In this work, we presented significant advancements toward fulfilling these criteria through the SO3LR model, which is embedded within an openly accessible and fully transparent framework. This framework integrates reliable and diverse quantum-mechanical data sets,^31,32,34,35,48^ a fast and stable SO3krates ML architecture,^39^ universal long-range interaction modules,^41^ a JAX-MD simulation engine,^37^ and robust analysis tools. Together, these components facilitate quantum-accurate molecular simulations across an extended chemical space.

Our developments aim toward enabling general molecular simulations and similar goals have been pursued by the seminal efforts in the empirical force field community over many decades.^80–88^ SO3LR achieves chemical accuracy and yields an 8-fold improvement compared to AmberFF for polyalanine^48^ when benchmarked on the PBE0+MBD atomic forces (Force MAE of 0.9 vs 7.6 kcal/mol/Å). At the same time, SO3LR is (only) about 40 times slower on a single GPU than GROMOS.^48^ Our extended assessment of energies, forces, dipoles, polarizabilities, as well as our analysis of nanosecond-long MD trajectories demonstrates that SO3LR is highly transferable throughout biochemical space and scalable to hundreds of thousands of atoms. Such transferability and scalability are achieved without the need to specify atom types, impose harmonic constraints, or introduce bespoke functional forms for interatomic interactions in different biomolecular entities. The bottom-up training on quantum mechanical data ensures that our simulations are transferable to a wider range of conditions than previously possible. This is confirmed by polyalanine simulations from 300 to 800 K, accurate structural and spectroscopic observables for high and low vibrational frequencies obtained for solvated crambin, as well as the local and global structural properties for the 1K7C glycoprotein and the POPC lipid bilayer. The toolset developed in this work complements the existing and quickly growing machinery of successful biomolecular modeling tools. Our presented advancements would not have been possible without building on a wealth of existing landmark methods, many of which were developed by the empirical force field community.

One noteworthy component of our proposed SO3LR model is a successful combination of explicit physical knowledge, such as short- and long-range force modules, coupled with a semilocal many-body potential. Importantly, all of these contributions are carefully balanced by SO3LR via learning from data. Thus, the known physical interactions do not need to be learned from data, but SO3LR can – under the correct hard-coded inductive biases (repulsion, electrostatics, and dispersion energies) – focus its nonlinear expressive power mainly on learning both the complex many-body contributions and the appropriate balance of the diverse energy terms from eq 1.

Despite recent progress in establishing “foundation models” for atomistic systems,^64,70,89,90^ many challenges remain in achieving truly general molecular simulations. While the SO3LR model shows broad applicability, it has several limitations that may guide future work. The model’s predictive accuracy is tied to its training data, possibly leading to suboptimal performance for underrepresented chemical environments such as curved carbon systems and specific functional groups (notably, dynamical simulations remain stable). Therefore, development of robust uncertainty quantification to reliably detect when the model is far in the extrapolation regime is a priority. Similarly, incorporating large-scale quantum mechanical data sets covering over 80 elements (e.g., QCML,^91^ MPTrj,^92^ and OMol25 ^93^), as well as expanding DFT+MBD training sets to include a broader spectrum of (bio)­chemical entities such as ions, sugars, lipids, DNA, supramolecules and diverse solvents would be highly beneficial for developing more transferable models applicable to (bio)­molecules, materials and their interfaces. Furthermore, future versions of SO3LR will integrate particle-mesh Ewald (PME) summation for long-range electrostatics, leveraging recent implementations in JAX-MD.^94^ Several other key areas for enhancing the SO3LR model include: (i) generating higher-level coupled cluster^95^ or quantum Monte Carlo^96^ reference data for small fragments, (ii) refining long-range interaction modules to effectively account for anisotropic many-body interactions,^97^ (iii) optimizing SO3LR for multi-GPU architectures,^98^ (iv) extending simulations to treat nuclear quantum effects^99,100,101^ beyond classical Newtonian MD. This is a nonexhaustive list of research directions, all of which are subjects of ongoing efforts in the community.

As atomistic simulations are highly sensitive to the intricacies of the underlying force fields and simulation parameters, it is imperative to establish a standardized set of benchmarks for quantum-accurate MLFFs. Such benchmarks will ensure reproducibility of results and enable robust modeling of experimentally relevant phenomena across realistic time and length scales.

## Supplementary Material



## Data Availability

The quantum mechanical data sets used for training and testing SO3LR are available at https://zenodo.org/records/14779793.^101^ The model and code used in this work are available at https://github.com/general-molecular-simulations/so3lr. The repository contains a convenient command-line interface and notebooks with tutorial simulations.

## Notes

Details of reference calculations; SO3krates architecture with compact introduction to invariance and equivariance; Ziegler-Biersack-Littmark inspired repulsion potential; charge equilibration procedure; long-range cutoff shifting scheme; SO3LR model training protocol; binding energy evaluation; molecular simulation setup and parameters; additional data set, model, and simulation analyses (PDF)

## References

[1] Schrödinger, E. What is Life? The Physical Aspect of the Living Cell; Cambridge University Press, 1974.

[2] Dirac P. A. M. (1929). Quantum
mechanics of many-electron systems. Proc. R.
Soc. London A.

[3] Feynman, R. P. , Leighton, R. B. , Sands, M. The Feynman lectures on physics, Vol. I: The new millennium ed.: mainly mechanics, radiation, and heat; Basic books: 2015.

[4] Huang B., von Rudorff G. F., von Lilienfeld O. A. (2023). The central role of density functional
theory in the AI age. Science.

[5] Dauber-Osguthorpe P., Hagler A. T. (2019). Biomolecular force
fields: where have we been, where
are we now, where do we need to go and how do we get there?. J. Comput. Aided Mol. Des..

[6] Hagler A. T. (2019). Force field
development phase II: Relaxation of physics-based criteria... or inclusion
of more rigorous physics into the representation of molecular energetics. J. Comput. Aided Mol. Des..

[7] MacKerell A. D. (2004). Empirical force fields for biological
macromolecules:
overview and issues. J. Comput. Chem..

[8] Van
Gunsteren W. F., Bakowies D., Baron R., Chandrasekhar I., Christen M., Daura X., Gee P., Geerke D. P., Glättli A., Hünenberger P.
H. (2006). Biomolecular
modeling: goals, problems, perspectives. Angew.
Chem., Int. Ed..

[9] Schlick T., Portillo-Ledesma S. (2021). Biomolecular
modeling thrives in the age of technology. Nat.
Comput. Sci..

[10] Noé F., Tkatchenko A., Müller K.-R., Clementi C. (2020). Machine learning for
molecular simulation. Annu. Rev. Phys. Chem..

[11] Unke O. T., Chmiela S., Sauceda H. E., Gastegger M., Poltavsky I., Schütt K. T., Tkatchenko A., Müller K.-R. (2021). Machine learning force fields. Chem. Rev..

[12] Poltavsky I., Tkatchenko A. (2021). Machine learning
force fields: Recent advances and
remaining challenges. J. Phys. Chem. Lett..

[13] Schütt, K. T. , Chmiela, S. , Von Lilienfeld, O. A. , Tkatchenko, A. , Tsuda, K. , Müller, K.-R. Machine Learning Meets Quantum Physics; Lecture Notes in Physics; Springer: 2020.

[14] Keith J. A., Vassilev-Galindo V., Cheng B., Chmiela S., Gastegger M., Muller K.-R., Tkatchenko A. (2021). Combining machine learning and computational
chemistry for predictive insights into chemical systems. Chem. Rev..

[15] Behler J., Parrinello M. (2007). Generalized
neural-network representation of high-dimensional
potential-energy surfaces. Phys. Rev. Lett..

[16] Bartók A. P., Payne M. C., Kondor R., Csányi G. (2010). Gaussian Approximation
Potentials: the accuracy of quantum mechanics, without the electrons. Phys. Rev. Lett..

[17] Behler J. (2011). Atom-centered
symmetry functions for constructing high-dimensional neural network
potentials. J. Chem. Phys..

[18] Schütt K. T., Sauceda H. E., Kindermans P.-J., Tkatchenko A., Müller K.-R. (2018). SchNet - a deep learning architecture for molecules
and materials. J. Chem. Phys..

[19] Unke O. T., Meuwly M. (2019). PhysNet: A neural network
for predicting energies,
forces, dipole moments, and partial charges. J. Chem. Theory Comput..

[20] Christensen A. S., Bratholm L. A., Faber F. A., Anatole von Lilienfeld O. (2020). FCHL revisited:
faster and more accurate quantum machine learning. J. Chem. Phys..

[21] Unke O. T., Chmiela S., Gastegger M., Schütt K. T., Sauceda H. E., Müller K.-R. (2021). SpookyNet:
Learning force fields
with electronic degrees of freedom and nonlocal effects. Nat. Commun..

[22] Frank T., Unke O., Müller K.-R. (2022). So3krates:
Equivariant attention
for interactions on arbitrary length-scales in molecular systems. Adv. Neural Inf. Process. Syst..

[23] Batzner S., Musaelian A., Sun L., Geiger M., Mailoa J. P., Kornbluth M., Molinari N., Smidt T. E., Kozinsky B. (2022). E­(3)-equivariant
graph neural networks for data-efficient and accurate interatomic
potentials. Nat. Commun..

[24] Batatia I., Kovacs D. P., Simm G., Ortner C., Csányi G. (2022). MACE: Higher
order equivariant message passing neural networks for fast and accurate
force fields. Adv. Neural Inf. Process. Syst..

[25] Anstine D. M., Isayev O. (2023). Machine learning interatomic potentials
and long-range
physics. J. Phys. Chem. A.

[26] Illarionov A., Sakipov S., Pereyaslavets L., Kurnikov I. V., Kamath G., Butin O., Voronina E., Ivahnenko I., Leontyev I., Nawrocki G. (2023). Combining
force fields
and neural networks for an accurate representation of chemically diverse
molecular interactions. J. Am. Chem. Soc..

[27] Blum L. C., Reymond J.-L. (2009). 970 million druglike
small molecules for virtual screening
in the chemical universe database gdb-13. J.
Am. Chem. Soc..

[28] Ramakrishnan R., Dral P. O., Rupp M., Von Lilienfeld O. A. (2014). Quantum
chemistry structures and properties of 134 kilo molecules. Sci. Data.

[29] Smith J. S., Isayev O., Roitberg A. E. (2017). ANI-1,
a data set of 20 million calculated
off-equilibrium conformations for organic molecules. Sci. Data.

[30] Smith J. S., Zubatyuk R., Nebgen B., Lubbers N., Barros K., Roitberg A. E., Isayev O., Tretiak S. (2020). The ANI-1ccx and ANI-1x
data sets, coupled-cluster and density functional theory properties
for molecules. Sci. Data.

[31] Donchev A. G., Taube A. G., Decolvenaere E., Hargus C., McGibbon R. T., Law K.-H., Gregersen B. A., Li J.-L., Palmo K., Siva K. (2021). Quantum
chemical benchmark databases of gold-standard
dimer interaction energies. Sci. Data.

[32] Hoja J., Medrano Sandonas L., Ernst B. G., Vazquez-Mayagoitia A., DiStasio R. A., Tkatchenko A. (2021). QM7-X, a comprehensive
dataset of quantum-mechanical properties spanning the chemical space
of small organic molecules. Sci. Data.

[33] Isert C., Atz K., Jiménez-Luna J., Schneider G. (2022). QMugs, quantum
mechanical properties of drug-like molecules. Sci. Data.

[34] Eastman P., Behara P. K., Dotson D. L., Galvelis R., Herr J. E., Horton J. T., Mao Y., Chodera J. D., Pritchard B. P., Wang Y., De Fabritiis G., Markland T. E. (2023). SPICE,
a dataset of drug-like molecules and peptides for training machine
learning potentials. Sci. Data.

[35] Medrano
Sandonas L., Van Rompaey D., Fallani A., Hilfiker M., Hahn D., Perez-Benito L., Verhoeven J., Tresadern G., Kurt Wegner J., Ceulemans H., Tkatchenko A. (2024). Dataset for quantum-mechanical exploration
of conformers and solvent effects in large drug-like molecules. Sci. Data.

[36] Eastman P., Swails J., Chodera J. D., McGibbon R. T., Zhao Y., Beauchamp K. A., Wang L.-P., Simmonett A. C., Harrigan M. P., Stern C. D. (2017). OpenMM 7: Rapid development
of high performance algorithms for molecular dynamics. PLOS Comput. Biol..

[37] Schoenholz S., Cubuk E. D. (2020). JAX MD: a framework for differentiable
physics. Adv. Neural Inf. Process. Syst..

[38] Pelaez R. P., Simeon G., Galvelis R., Mirarchi A., Eastman P., Doerr S., Thölke P., Markland T. E., De Fabritiis G. (2024). TorchMD-Net
2.0: Fast neural network potentials for molecular simulations. J. Chem. Theory Comput..

[39] Frank J. T., Unke O. T., Müller K.-R., Chmiela S. (2024). A euclidean transformer
for fast and stable machine learned force fields. Nat. Commun..

[40] Ziegler, J. F. , Biersack, J. P. , Littmark, U. The stopping and range of ions in solids; Pergamon Press: New York, 1985.

[41] Khabibrakhmanov A., Fedorov D. V., Tkatchenko A. (2023). Universal
pairwise interatomic van
der waals potentials based on quantum drude oscillators. J. Chem. Theory Comput..

[42] Hirshfeld F. L. (1977). Bonded-atom
fragments for describing molecular charge densities. Theor. Chim. Acta.

[43] Johnson E.
R., Becke A. D. (2006). A post-hartree-fock
model of intermolecular interactions:
Inclusion of higher-order corrections. J. Chem.
Phys..

[44] Fedorov D. V., Sadhukhan M., Stöhr M., Tkatchenko A. (2018). Quantum-mechanical
relation between atomic dipole polarizability and the van der waals
radius. Phys. Rev. Lett..

[45] Tkatchenko A., Scheffler M. (2009). Accurate molecular
van der waals interactions from
ground-state electron density and free-atom reference data. Phys. Rev. Lett..

[46] Jones A. P., Crain J., Sokhan V. P., Whitfield T. W., Martyna G. J. (2013). Quantum drude oscillator model of atoms and molecules:
Many-body polarization and dispersion interactions for atomistic simulation. Phys. Rev. B.

[47] Rezác J., Riley K. E., Hobza P. (2011). S66: A well-balanced database of
benchmark interaction energies relevant to biomolecular structures. J. Chem. Theory Comput..

[48] Unke O. T., Stöhr M., Ganscha S., Unterthiner T., Maennel H., Kashubin S., Ahlin D., Gastegger M., Medrano Sandonas L., Berryman J. T. (2024). Biomolecular dynamics
with machine-learned quantum-mechanical force fields trained on diverse
chemical fragments. Sci. Adv..

[49] Adamo C., Barone V. (1999). Toward reliable density
functional methods without
adjustable parameters: The PBE0 model. J. Chem.
Phys..

[50] Tkatchenko A., DiStasio R. A., Car R., Scheffler M. (2012). Accurate and
efficient method for many-body van der waals interactions. Phys. Rev. Lett..

[51] Baldauf C., Rossi M. (2015). Going clean: structure and dynamics of peptides in the gas phase
and paths to solvation. J. Condens. Matter Phys..

[52] Schubert F., Rossi M., Baldauf C., Pagel K., Warnke S., von Helden G., Filsinger F., Kupser P., Meijer G., Salwiczek M. (2015). Exploring the conformational preferences of
20-residue peptides in isolation: Ac-Ala19-Lys + H+ vs. Ac-Lys-Ala19
+ H+ and the current reach of DFT. Phys. Chem.
Chem. Phys..

[53] Al-Hamdani Y. S., Nagy P. R., Zen A., Barton D., Kállay M., Brandenburg J. G., Tkatchenko A. (2021). Interactions between large molecules
pose a puzzle for reference quantum mechanical methods. Nat. Commun..

[54] Hoja J., Ko H.-Y., Neumann M. A., Car R., DiStasio R. A., Tkatchenko A. (2019). Reliable and
practical computational
description of molecular crystal polymorphs. Sci. Adv..

[55] Firaha D., Liu Y. M., van de
Streek J., Sasikumar K., Dietrich H., Helfferich J., Aerts L., Braun D. E., Broo A., DiPasquale A. G. (2023). Predicting crystal form
stability under real-world conditions. Nature.

[56] Huang B., von Lilienfeld O. A. (2020). Quantum
machine learning using atom-in-molecule-based
fragments selected on the fly. Nat. Chem..

[57] Charkin-Gorbulin A., Kokorin A., Sauceda H. E., Chmiela S., Quarti C., Beljonne D., Tkatchenko A., Poltavsky I. (2025). Atomic orbits
in molecules and materials for improving machine learning force fields.. Mach. Learn.: Sci. Technol..

[58] Staacke C. G., Wengert S., Kunkel C., Csányi G., Reuter K., Margraf J. T. (2022). Kernel charge equilibration: efficient
and accurate prediction of molecular dipole moments with a machine-learning
enhanced electron density model. Mach. Learn.:
Sci. Technol..

[59] Chmiela S., Vassilev-Galindo V., Unke O. T., Kabylda A., Sauceda H. E., Tkatchenko A., Müller K.-R. (2023). Accurate global machine learning
force fields for molecules with hundreds of atoms. Sci. Adv..

[60] Yang Y., Lao K. U., Wilkins D. M., Grisafi A., Ceriotti M., DiStasio R. A. (2019). Quantum mechanical static dipole
polarizabilities in the QM7b and AlphaML showcase databases. Sci. Data.

[61] Villot C., Lao K. U. (2024). Ab initio dispersion
potentials based on physics-based
functional forms with machine learning. J. Chem.
Phys..

[62] Rai B. K., Sresht V., Yang Q., Unwalla R., Tu M., Mathiowetz A. M., Bakken G. A. (2022). TorsionNet: A deep neural network
to rapidly predict small-molecule torsional energy profiles with the
accuracy of quantum mechanics. J. Chem. Inf.
Model..

[63] Lovell S. C., Davis I. W., Arendall W. B., De
Bakker P. I., Word J. M., Prisant M. G., Richardson J. S., Richardson D. C. (2003). Structure validation by C*
_α_
* geometry: *ϕ*, *ψ* and C*
_β_
* deviation. Proteins Struct. Funct. Bioinform..

[64] Kovács D. P., Moore J. H., Browning N. J., Batatia I., Horton J. T., Pu Y., Kapil V., Witt W. C., Magdău I.-B., Cole D. J. (2025). MACE-OFF: Short-range transferable machine learning
force fields for organic molecules. J. Am. Chem.
Soc..

[65] Millhauser G. L., Stenland C. J., Hanson P., Bolin K. A., van de
Ven F. J. (1997). Estimating the relative populations of 3_10_-helix and *α*-helix in ala-rich peptides: a hydrogen exchange
and high field NMR study. J. Mol. Biol..

[66] Bolin K. A., Millhauser G. L. (1999). *α* and 3_10_: the split
personality of polypeptide helices. Acc. Chem.
Res..

[67] Ahn H.-C., Juranić N., Macura S., Markley J. L. (2006). Three-dimensional
structure of the water-insoluble protein crambin in dodecylphosphocholine
micelles and its minimal solvent-exposed surface. J. Am. Chem. Soc..

[68] McInnes, L. , Healy, J. , Melville, J. UMAP: Uniform manifold approximation and projection for dimension reduction, arXiv preprint 10.48550/arXiv.1802.03426 (2018).

[69] Ferreira T. M., Coreta-Gomes F., Ollila O. S., Moreno M. J., Vaz W. L., Topgaard D. (2013). Cholesterol and POPC segmental order parameters in
lipid membranes: solid state ^1^H-^13^C NMR and
MD simulation studies. Phys. Chem. Chem. Phys..

[70] Anstine D. M., Zubatyuk R., Isayev O. (2025). AIMNet2: a
neural network potential
to meet your neutral, charged, organic, and elemental-organic needs. Chem. Sci..

[71] Kohtani M., Jones T. C., Schneider J. E., Jarrold M. F. (2004). Extreme stability
of an unsolvated *α*-helix. J. Am. Chem. Soc..

[72] DiStasio R. A., Santra B., Li Z., Wu X., Car R. (2014). The individual
and collective effects of exact exchange and dispersion interactions
on the ab initio structure of liquid water. J. Chem. Phys..

[73] Soper A. K. (2013). The radial
distribution functions of water as derived from radiation total scattering
experiments: is there anything we can say for sure?. Int. Sch. Res. Notices.

[74] Mills R. (1973). Self-diffusion
in normal and heavy water in the range 1–45. deg. J. Phys. Chem..

[75] Guvench O., Mallajosyula S. S., Raman E. P., Hatcher E., Vanommeslaeghe K., Foster T. J., Jamison F. W., MacKerell A. D. (2011). CHARMM additive all-atom force field for carbohydrate
derivatives and its utility in polysaccharide and carbohydrate-protein
modeling. J. Chem. Theory Comput..

[76] Kučerka N., Nieh M.-P., Katsaras J. (2011). Fluid phase
lipid areas and bilayer
thicknesses of commonly used phosphatidylcholines as a function of
temperature. Biochim. Biophys. Acta.

[77] Dickson C. J., Walker R. C., Gould I. R. (2022). Lipid21:
complex lipid membrane simulations
with Amber. J. Chem. Theory Comput..

[78] Yu Y., Kramer A., Venable R. M., Brooks B. R., Klauda J. B., Pastor R. W. (2021). CHARMM36 lipid force
field with explicit treatment
of long-range dispersion: parametrization and validation for phosphatidylethanolamine,
phosphatidylglycerol, and ether lipids. J. Chem.
Theory Comput..

[79] Wang Y., Takaba K., Chen M. S., Wieder M., Xu Y., Zhu T., Zhang J. Z., Nagle A., Yu K., Wang X. (2025). On the
design space between molecular mechanics and machine learning
force fields. Appl. Phys. Rev..

[80] Case D. A., Cheatham T. E., Darden T., Gohlke H., Luo R., Merz K. M., Onufriev A., Simmerling C., Wang B., Woods R. J. (2005). The Amber biomolecular simulation
programs. J. Comput. Chem..

[81] Wang J., Wolf R. M., Caldwell J. W., Kollman P. A., Case D. A. (2004). Development
and testing of a general Amber force field. J. Comput. Chem..

[82] Brooks B. R., Brooks C. L., Mackerell A. D., Nilsson L., Petrella R. J., Roux B., Won Y., Archontis G., Bartels C., Boresch S. (2009). CHARMM:
the biomolecular simulation program. J. Comput.
Chem..

[83] Vanommeslaeghe K., Hatcher E., Acharya C., Kundu S., Zhong S., Shim J., Darian E., Guvench O., Lopes P., Vorobyov I. (2010). CHARMM
general force field: A force field for
drug-like molecules compatible with the charmm all-atom additive biological
force fields. J. Comput. Chem..

[84] Zhu X., Lopes P. E., MacKerell A. D. (2012). Recent developments
and applications of the CHARMM force fields. Wiley Interdiscip. Rev. Comput. Mol. Sci..

[85] Jorgensen W. L., Maxwell D. S., Tirado-Rives J. (1996). Development and testing of the OPLS
all-atom force field on conformational energetics and properties of
organic liquids. J. Am. Chem. Soc..

[86] Oostenbrink C., Villa A., Mark A. E., Van Gunsteren W. F. (2004). A biomolecular
force field based on the free enthalpy of hydration and solvation:
the GROMOS force-field parameter sets 53a5 and 53a6. J. Comput. Chem..

[87] Schmid N., Eichenberger A. P., Choutko A., Riniker S., Winger M., Mark A. E., Van Gunsteren W. F. (2011). Definition and testing of the GROMOS
force-field versions 54a7 and 54b7. Eur. Biophys.
J..

[88] Ponder J. W., Wu C., Ren P., Pande V. S., Chodera J. D., Schnieders M. J., Haque I., Mobley D. L., Lambrecht D. S., DiStasio R. A. (2010). Current status of the
AMOEBA polarizable force field. J. Phys. Chem.
B.

[89] Zubatyuk R., Smith J. S., Leszczynski J., Isayev O. (2019). Accurate and transferable
multitask prediction of chemical properties with an atoms-in-molecules
neural network. Sci. Adv..

[90] Plé T., Lagardère L., Piquemal J.-P. (2023). Force-field-enhanced neural network
interactions: from local equivariant embedding to atom-in-molecule
properties and long-range effects. Chem. Sci..

[91] Ganscha S., Unke O. T., Ahlin D., Maennel H., Kashubin S., Müller K.-R. (2025). The QCML
dataset, quantum chemistry reference data
from 33.5M DFT and 14.7B semi-empirical calculations. Sci. Data.

[92] Deng B., Zhong P., Jun K., Riebesell J., Han K., Bartel C. J., Ceder G. (2023). CHGNet as
a pretrained universal
neural network potential for charge-informed atomistic modelling. Nat. Mach. Intell..

[93] Levine, D. S. , Shuaibi, M. , Spotte-Smith, E. W. C. , Taylor, M. G. , Hasyim, M. R. , Michel, K. , Batatia, I. , Csányi, G. , Dzamba, M. , Eastman, P. The open molecules 2025 (OMol25) dataset, evaluations, and models, arXiv preprint 10.48550/arXiv.2505.08762 (2025).

[94] Loche P., Huguenin-Dumittan K. K., Honarmand M., Xu Q., Rumiantsev E., How W. B., Langer M. F., Ceriotti M. (2025). Fast and flexible long-range
models for atomistic machine learning. J. Chem.
Phys..

[95] Chmiela S., Sauceda H. E., Müller K.-R., Tkatchenko A. (2018). Towards exact
molecular dynamics simulations with machine-learned force fields. Nat. Commun..

[96] Slootman E., Poltavsky I., Shinde R., Cocomello J., Moroni S., Tkatchenko A., Filippi C. (2024). Accurate quantum monte
carlo forces for machine-learned force fields: Ethanol as a benchmark. J. Chem. Theory Comput..

[97] Stöhr M., Tkatchenko A. (2019). Quantum mechanics of proteins in explicit water: The
role of plasmon-like solute-solvent interactions. Sci. Adv..

[98] Park Y., Kim J., Hwang S., Han S. (2024). Scalable parallel algorithm for graph
neural network interatomic potentials in molecular dynamics simulations. J. Chem. Theory Comput..

[99] Sauceda H. E., Vassilev-Galindo V., Chmiela S., Müller K.-R., Tkatchenko A. (2021). Dynamical strengthening of covalent and non-covalent
molecular interactions by nuclear quantum effects at finite temperature. Nat. Commun..

[100] Musil F., Zaporozhets I., Noé F., Clementi C., Kapil V. (2022). Quantum dynamics using
path integral
coarse-graining. J. Chem. Phys..

[101] Kabylda, A. , Frank, J. T. , Dou, S. S. , Khabibrakhmanov, A. , Sandonas, L. M. , Unke, O. T. , Chmiela, S. , Müller, K.-R. , Tkatchenko, A. DFT data for “molecular simulations with a pretrained neural network and universal pairwise force fields”, 10.5281/zenodo.14779793 (2025). PMC12447504 40886167

