# Supplementary material for: Molecular Simulations with a Pretrained Neural Network and Universal Pairwise Force Fields
Source: J Am Chem Soc. 2025 Aug 31;147(37):33723–34. doi: 10.1021/jacs.5c09558 (PMC12447504; doi:10.1021/jacs.5c09558)
Supplement: Supplementary file 1 [file ja5c09558_si_001.pdf]

Supporting Information
Molecular Simulations with a Pretrained Neural Network and Universal Pairwise Force Fields

Adil Kabylda[‖,1,*] J. Thorben Frank[‖,2,3,†] Sergio Suárez-Dou,[1] Almaz Khabibrakhmanov,[1] Leonardo Medrano Sandonas,[4] Oliver T. Unke,[5] Stefan Chmiela,[2,3] Klaus-Robert Müller,[2,3,5,6,7,‡] and Alexandre Tkatchenko[1,§]

[1] *Department of Physics and Materials Science, University of Luxembourg, L-1511 Luxembourg City, Luxembourg*
[2] *Machine Learning Group, Technische Universität Berlin, 10587 Berlin, Germany*
[3] *Berlin Institute for the Foundations of Learning and Data – BIFOLD, 10587 Berlin, Germany*
[4] *Institute for Materials Science and Max Bergmann Center of Biomaterials, TUD Dresden University of Technology, 01069 Dresden, Germany*
[5] *Google DeepMind, 10117 Berlin, Germany*
[6] *Max Planck Institute for Informatics, Stuhlsatzenhausweg, 66123 Saarbrücken, Germany*
[7] *Department of Artificial Intelligence, Korea University, Anam-dong, Seongbuk-gu, 02841 Seoul, Korea*

MATERIALS AND METHODS

Reference calculations. All reference calculations were performed at the PBE0+MBD level of theory using the FHI-aims code [1, 2]. "Tight" settings were applied for basis functions and integration grids. Energies were converged to 10^{-6} eV and the force accuracy was set to 10^{-5} eV/Å. The self-consistent field (SCF) optimization convergence criteria were 10^{-5} eV for the sum of eigenvalues and 10^{-6} electrons/Å^3 for the charge density. The Zenodo repository contains the relevant settings file.

SO3krates. The SO3krates neural network contains two sets of features: High-dimensional invariant atomic features $\mathcal{H} = \{\boldsymbol{h}_1, \ldots, \boldsymbol{h}_N \,|\, \boldsymbol{h}_i \in \mathbb{R}^H\}$ and low-dimensional equivariant atomic features $\mathcal{X} = \{\boldsymbol{x}_1, \ldots, \boldsymbol{x}_N \,|\, \boldsymbol{x}_i \in \mathbb{R}^{(L+1)^2}\}$, where L denotes the maximal degree of the spherical harmonic used in the network. For a compact and formal introduction into invariance and equivariance see subsection "Symmetry and Equivariance".

Initial invariant atomic features encode information about the atomic types Z, the total charge Q and the multiplicity S of the system. Whereas the atomic types are defined for each atom in the system, the total charge and multiplicity is defined per molecule. On a high level initial features are calculated as

$$\boldsymbol{h}_i^{[0]} = \boldsymbol{e}_{i,Z} + \boldsymbol{e}_{i,Q} + \boldsymbol{e}_{i,S}, \tag{S1}$$

where each summand is a H-dimensional embedding vector for each atom i in the system.

Atomic numbers are encoded as

$$\boldsymbol{e}_{i,Z} = \mathrm{Embed}(z_i), \tag{S2}$$

where "Embed" is an embedding function that takes an atomic number $z_i \in \mathbb{N}_+$ and returns a H-dimensional embedding vector.

Following the strategy described in Ref. [3], total charge and multiplicity are encoded as

$$\boldsymbol{p}_i = \mathrm{Embed}(Z_i), \quad \boldsymbol{k} = \begin{cases} \boldsymbol{k}_+ & \text{if } \Psi \geq 0 \\ \boldsymbol{k}_- & \text{if } \Psi < 0 \end{cases} \quad \boldsymbol{v} = \begin{cases} \boldsymbol{v}_+ & \text{if } \Psi \geq 0 \\ \boldsymbol{v}_- & \text{if } \Psi < 0 \end{cases}$$
$$a_i = \frac{\Psi \ln\left(1 + \exp \boldsymbol{p}_i^\intercal \cdot \boldsymbol{k}/\sqrt{H}\right)}{\sum_{j=1}^N \ln\left(1 + \exp \boldsymbol{p}_j^\intercal \cdot \boldsymbol{k}/\sqrt{H}\right)}, \quad \boldsymbol{e}_{i,\Psi} = \mathrm{MLP}(a_i \boldsymbol{v}_i), \tag{S3}$$

where $\boldsymbol{k}, \boldsymbol{v} \in \mathbb{R}^H$ are trainable parameters, and "Embed" is another embedding function for atomic numbers and "MLP" is a two-layered multi-layer perceptron (MLP) network. Separate parameters are used for charge $\Psi = Q$ and spin $\Psi = S$ embeddings and also for positive and negative values of Ψ, indicated by the subscripts "+" and "−", respectively. Since S is always equal or larger than zero, only the positive terms are used. Additionally, the MLP does not use any bias terms, such that $\mathrm{MLP}(a\boldsymbol{v}) = \boldsymbol{0}$ if $a\boldsymbol{v} = \boldsymbol{0}$. The described encoding procedure globally distributes the information about the total charge and the spin state, using per-atom weighting factors a_i.

The low-dimensional equivariant features are initialized to all-zeros i.e., $\boldsymbol{x} = \boldsymbol{0}$. This ensures that equivariance is preserved throughout the network. Alternative embedding schemes could be employed to embed equivariant features, i.e., an initial neighborhood scan as done in the original publications or in other equivariant MPNN approaches [4, 5].

After creating initial atomic features $(\mathcal{H}^{[t=0]}, \mathcal{X}^{[t=0]})$ they are iteratively refined via T fast equivariant MP layers as

$$\left(\mathcal{H}_i^{[t+1]}, \mathcal{X}_i^{[t+1]}\right) = \mathrm{FastEquivMP}\left[\mathcal{H}^{[t]}, \mathcal{X}^{[t]}, \mathcal{G}_\mathcal{R}\right], \tag{S4}$$

where $\mathcal{G}_\mathcal{R} = (\mathcal{R}, \mathcal{E})$ denotes a geometric graph, containing information about the atomic positions $\mathcal{R}$ and the inter-atomic connectivity via "Edges" $\mathcal{E}$. Edges are determined based on a local cutoff radius r_{cut} around each

* adil.kabylda@uni.lu
† thorbenjan.frank@gmail.com
‡ klaus-robert.mueller@tu-berlin.de
§ alexandre.tkatchenko@uni.lu
‖ These authors contributed equally to this work

atom, and atoms lying within the cutoff sphere are considered a neighbor of the central atom, i.e., they share an edge. Here we use a cutoff of $r_{\text{cut}} = 4.5\,\text{Å}$, greatly exceeding covalent bond lengths. In contrast to classical FFs, which often assume a fixed connectivity, a geometric graph is re-constructed for every set of atomic positions, such that breaking and forming of atomic bonds is handled naturally.

Each layer "FastEquivMP" layer consists of two phases. In the first phase, information from neighboring atoms is aggregated. In the second phase, the high-dimensional invariant and the low-dimensional equivariant features exchange information on a per-atom basis. This design ensures low computational cost while maintaining the benefits of equivariant feature representations. For full information the reader is referred to the original publication in Ref. [5].

The final invariant features $\boldsymbol{h}_i^{[T]} \in \mathbb{R}^H$ are used to predict the total energy of the molecule as

$$E_{\text{SO3k}} = \sum_{i=1}^{N} \text{MLP}\Big(\boldsymbol{h}_i^{[T]}\Big), \tag{S5}$$

with a two-layer MLP outputing a scalar energy contribution for each atom in the molecule. Forces are obtained as the gradient w.r.t. atomic positions $\vec{F}_i = -\nabla_{\vec{r}_i} E_{\text{SO3k}}$ using automatic differentation.

Symmetry and Equivariance. A group G is a set equipped with a binary operation known as multiplication, which fulfills the axioms of *associativity*, *identity*, *closure* and *inverse*. Given an abstract group G with elements g, a function $f : \mathcal{X} \mapsto \mathcal{Y}$ between sets $\mathcal{X}$ and $\mathcal{Y}$ is equivariant *w.r.t.* to G if

$$f(g * x) = g * f(x)\,, \tag{S6}$$

where "$*$" denotes the group action.

If the functions operate on vectors (e.g. atomic positions or high-dimensional learned representations) one needs to represent the abstract group in terms of real vector spaces. Therefore, lets assume a d-dimensional vector space X with elements $\boldsymbol{x} \in X$. A *group representation* of G on $X = \mathbb{R}^d$ is given as a continuous function $\rho^X : G \mapsto \mathbb{R}^{d\times d}$ from the group to real square invertible matrices, such that for all $g, h \in G$ the following relation holds

$$\rho^X(g)\rho^X(h) = \rho^X(g \odot h). \tag{S7}$$

This means, that the group operation "$\odot$" can be represented in terms of multiplications between matrices and allows to give a definition of equivariance for vector-valued functions.

Finally, a function $f : X \mapsto Y$ between vector spaces X and Y is said to be equivariant *w.r.t.* to G if for all $g \in G$ it holds that

$$f\big(\rho^X(g)\boldsymbol{x}\big) = \rho^Y(g) f(\boldsymbol{x}), \tag{S8}$$

where $\rho^X(g)$ and $\rho^Y(g)$ are group representations on X and Y, respectively. This makes *invariance* a special case of equivariance with $\rho^Y(g) = \boldsymbol{I}$ being the identity matrix in Y.

In our context, we typically consider the group of rotations $G = \text{SO}(3)$, such that the group elements g are rotation matrices $\boldsymbol{M}_{\text{rot}} \in \mathbb{R}^{3\times 3}$. When we talk about equivariance (invariance) we implicitly refer to equivariance (invariance) *w.r.t.* rotations of the input atomic positions. As an example for invariance, lets consider the potential energy surface (PES), which is a function $U : \mathbb{R}^{N\times 3} \mapsto \mathbb{R}$ and is invariant *w.r.t.* rotations of the atomic positions. In the formalism described above, this corresponds to X being the Euclidean space of 3 dimensions, the group representations on X are the rotation matrices i.e. $\rho^X(g) = \boldsymbol{M}_{\text{rot}} \in \mathbb{R}^{3\times 3}$ and the group representations on the output are $\rho^Y(g) = 1$. Therefore,

$$U(\boldsymbol{M}_{\text{rot}} R, Z) = U(R, Z), \tag{S9}$$

where $M_{\text{rot}} R$ denotes the global rotation of all atomic positions.

The force function for a single atom is given as $\vec{F}_i : \mathbb{R}^{N\times 3} \mapsto \mathbb{R}^3$. Under global rotations, this function behaves as

$$\vec{F}_i(\boldsymbol{M}_{\text{rot}} R) = \boldsymbol{M}_{\text{rot}} \vec{F}_i(R), \tag{S10}$$

which makes forces a rotationally equivariant function, with $\rho^Y(g)$ now also being rotation matrices.

In the context of MLFFs, we consider a neural network (NN) $f_{\text{NN}} : \mathbb{R}^{N\times 3} \mapsto \mathbb{R}^d$, mapping the input positions to a high-dimensional representation of dimension d. Equivariant NNs learn representations which behave according to Eq. S8. In this setting, invariant MLFFs like SchNet [6] or PhysNet [7] are a special case of equivariance and correspond to $\rho^Y(g) = \boldsymbol{I}$. In contrast, equivariant MLFFs like NequIP [8] construct representations which transform equivariant under rotation of the input positions and $\rho^Y(g)$ is a direct sum of Wigner-D matrices.

Ziegler-Biersack-Littmark repulsion. The short-range repulsion between nuclei is modeled via a term inspired by the ZBL repulsion [3, 9]:

$$\begin{aligned} E_{\text{ZBL}} = k_e \sum_i \sum_{j\in\mathcal{N}_i} \frac{Z_i Z_j}{r_{ij}} f_{\text{cut}}(r_{ij}) \\ \cdot \sum_{m=1}^{4} c_m e^{-a_m r_{ij}(Z_i^p + Z_j^p)/d}, \end{aligned} \tag{S11}$$

where k_e is the Coulomb constant, Z_i are the atomic numbers, and a_m, c_m, p, and d are free parameters. The term $\mathcal{N}_i$ denotes the neighborhood of the i-th atom, and f_{cut} is a cutoff function that smoothly transitions between one and zero when atoms leave (or enter) the neighborhood. The ZBL term ensures a correct description of nuclear repulsion, which improves the stability of the potential for short bond-distances.

Partial charges and Dipoles. Following Ref. 3, partial charges are obtained as

$$q_i = q_{Z_i} + \tilde{q}_i + \frac{1}{N}\left(Q - \sum\nolimits_{j=1}^{N}(q_{Z_j} + \tilde{q}_j)\right), \quad \text{(S12)}$$

where $\tilde{q}_i \in \mathbb{R}$ are predicted from the final atomic representations $\boldsymbol{h}_i^{[T]} \in \mathbb{R}^H$ via a two-layered MLP network with silu non-linearity and $q_{Z_i} \in \mathbb{R}$ is an element dependent bias. The charge correction with the total charge Q ensures charge conservation. The partial charges can be used to predict molecular dipole moments (used in the loss function, see Eq. 7).

$$\vec{\mu} = \sum_{i=1}^{N} q_i \vec{r}_i, \quad \text{(S13)}$$

where $\vec{r}_i \in \mathbb{R}^3$ are the atomic positions (assumed to be centered).

Long-range cutoff. For large structures with tens to hundreds of thousands of atoms, considering all pairs of atoms becomes computationally infeasible and necessitates the introduction of a long-range cutoff. Additionally, if simulations are performed in a box (e.g. with water) the largest meaningful long-range cutoff is directly connected to the box size. As such, the system under investigation and the simulation parameters, determine different values for the long-range cutoff. To account for this, we carefully designed a switching function for the long-range potentials, which allows to choose between different cutoff values up to no long-range cutoff at the time of simulation. The choice does not affect the first two terms in Eq. 1 or intermediate properties, partial charges and Hirshfeld ratios, which are used as inputs to calculate the last two terms.

Both the dispersion and the electrostatic potential have infinite range and take on a non-zero value at the long-range cutoff (Fig. S11). This results in a discontinuity in the forces at the cutoff value, leading to energy drift during MD simulations [10]. To ensure smoothness of the PES at the long-range cutoff we modify the pairwise electrostatic potential as

$$\tilde{u}(r) = \frac{q_i q_j k_e}{2} \cdot f_{\text{sw}}(r) \cdot u_{\text{ES}}(r) + \big(1 - f_{\text{sw}}(r)\big) \cdot u_{\text{FS}}(r), \quad \text{(S14)}$$

where $u_{\text{ES}}(r)$ is the energy-shifted potential, $u_{\text{FS}}(r)$ is the force-shifted potential (see below) and $f_{\text{sw}}(r)$ is a switching function that smoothly interpolates between 1 and 0 on a given interval from r_{on} to r_{off}. By switching between the energy- and the force-shifted terms, the potential remains unaltered within the short-range cutoff (which maintains the learned balance between different terms, as there was no long-range cutoff during training) while smoothly transitioning to zero at the long-range cutoff (Fig. S11). The shifted potentials are given as [10, 11]

$$u_{\text{ES}}(r) = \begin{cases} u(r) - u(R_c), & r < R_c \\ 0, & r > R_c \end{cases} \quad \text{(S15)}$$

and

$$u_{\text{FS}}(r) = \begin{cases} u(r) - u(R_c) - u(r - R_c) \cdot u'(R_c) & r < R_c \\ 0, & r > R_c \end{cases} \quad \text{(S16)}$$

where $u(r)$ is the unmodified pairwise electrostatic potential (Eq. 6) and R_c is the long-range cutoff. Dispersion interactions are smoothly switched to zero as

$$\tilde{v}(r) = f_{\text{sw}}(r) \cdot v(r), \quad \text{(S17)}$$

where $v(r)$ is the pairwise potential in Eq. 4. The switching function parameters $(r_{\text{on}}, r_{\text{off}})$ were set to $(R_c \times 0.45, R_c)$ for electrostatic interactions and $(R_c - 2, R_c)$ for dispersion interactions. The parameters were chosen to prevent clumping artifacts at the 10 Å long-range cutoff.

Training details. SO3krates model (v1.0) was trained on a combined loss of forces, dipole moments, and Hirshfeld ratios with a weighting factor of 10:1:1, respectively. We used the AMSGrad optimizer [12] with an initial learning rate of 10^{-3} and an exponential learning rate decay every 500k steps by a factor of 0.85. The global norm of the gradient updates is clipped at 10.

The model uses a 4.5 Å cutoff, feature dimension of $H = 128$, and a maximal degree of $L = 4$ for the Euclidean variables and $T = 3$ message passing layers, electrostatics damping coefficient of $\sigma = 4$, and dispersion damping coefficient of $\gamma = 1.2$. After each attention update, a two-layered multi-layer perceptron with silu non-linearity refines the invariant features. This increases the number of trainable parameters and thus model expressiveness, which is important in the large data regime. To stabilize training and improve gradient flow, layer normalization [13] is applied to the invariant features after the attention and the interaction block. The model was trained on a single A100 GPU for 86 h (corresponding to 5.125M gradient steps) with a batch size of $B = 200$.

Binding energy calculation. Binding energy was calculated as the difference between the bound dimer and the non-interacting monomers (separated by a distance larger than the long-range cutoff) with charges assigned for each monomer in isolation. The GitHub repository contains an example script, demonstrating the binding energy computation.

Simulation details. All simulations were conducted using the NVT ensemble for gas-phase systems (with a long-range cutoff of 100 Å) and the NPT ensemble for periodic systems (with a long-range cutoff of 12 Å), with a timestep of 0.5 fs. Nosé–Hoover Chains (3 chains) were used for thermostat and barostat coupling, as implemented in JAX-MD [14], with default parameters: 1000 timesteps for the barostat and 100 timesteps for the thermostat [15–17]. Prior to simulation, all structures were pre-optimized using the FIRE algorithm [18].

MD22 molecules and polyalanines. Gas-phase simulations for stachyose and $AcAla_3NMe$ were performed for 500 ps at 500 K, and for other MD22 molecules at 300 K. Simulations of $AcAla_{15}NMe$ folding were carried out at

300 K (with initial velocities sampled from a Maxwell-Boltzmann distribution at 600 K). $AcAla_{15}LysH^{+}$ was simulated at 500–800 K with a step of 100 K, each for 500 ps. Secondary structure assignment of polyalanines was performed using the STRIDE algorithm [19].

Water simulations. Simulations were run for 500 ps, with observables averaged over the final 300 ps. The diffusion coefficient was determined from the positions of oxygen atoms using Einstein diffusion equation [20, 21]. Double-precision was employed to enhance numerical stability.

Crambin. The initial structure was obtained from PDB ID: 2FD7 [22], with mutated residues reverted to the wild-type sequence. The system was solvated with 8205 explicit water molecules. Simulations were performed for 3 ns at 300 K, excluding the first 0.5 ns for equilibration. The root mean square deviation of Crambin, RMSD(t, t+Δt), was obtained excluding hydrogen atoms from three 3 ns runs. Power spectra were computed from atomic velocities sampled over a 125 ps trajectory with a time resolution of 2.5 fs using schnetpack package [23, 24].

Glycoprotein. The starting structure was taken from the PDB ID: 1K7C [25]. 15008 water molecules were used for solvating the system and the pH was set to 3.7 to guarantee charge neutralization. The RMSD was calculated based on three runs of 500 ps.

POPC Lipid bilayer. The starting structure, consisting of 128 lipids and 5120 water molecules, was obtained from Ref. 26. The system was equilibrated over 250 ps using a combination of geometry relaxations and NVT simulations. Observables were then averaged over an additional 250 ps at 303 K in an isotropic NPT ensemble implemented in JAX-MD. The initial box dimensions were adjusted manually to mimic semi-isotropic NPT ensemble. The area per lipid was calculated from the simulation box dimensions. Bilayer thickness (D_{HH}), derived from electron density profiles, and NMR order parameters were both calculated using CPPTRAJ [27]. Double-precision was employed to enhance numerical stability.

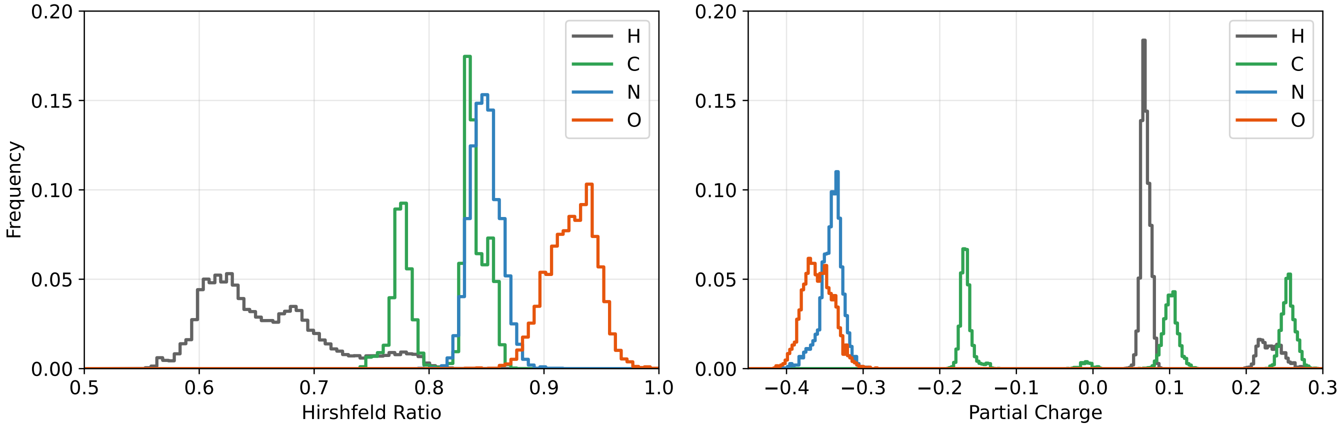

Fig. S1. Hirshfeld ratio and partial charge distribution for $AcAla_{15}NMe$. Both quantities are dynamically changing and are predicted by the SO3LR model.

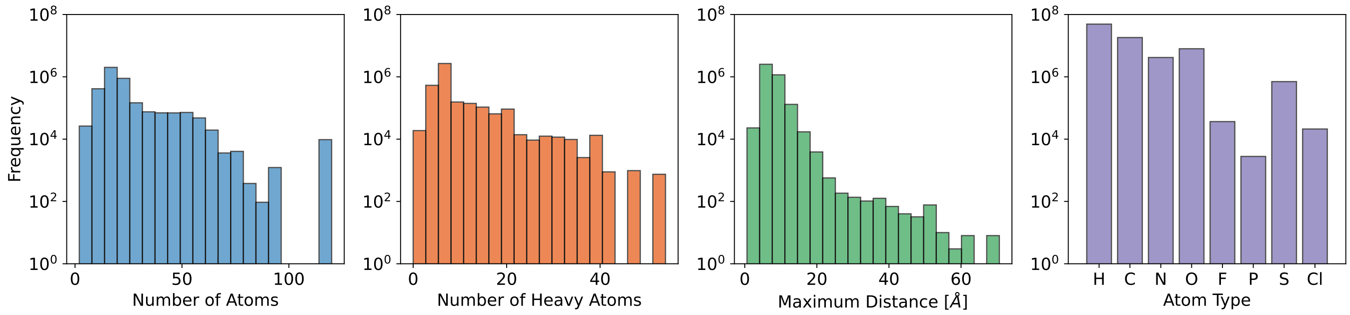

Fig. S2. Statistics on a combined dataset of 3.9 million molecular fragments. Histograms of the number of atoms, number of heavy atoms, maximum distance in each fragment, and atom types.

TABLE S1. Properties present in the combined datasets.

Dataset	Size	Forces	Dipoles	Hirsh. rat.
GEMS bottom-up	2.7m	✓	✓	✗
QM7-X	1m	✓	✓	✓
AQM	60k	✓	✓	✓
SPICE Dipeptides	33k	✓	✓	✓
DES15k	15k	✓	✓	✓

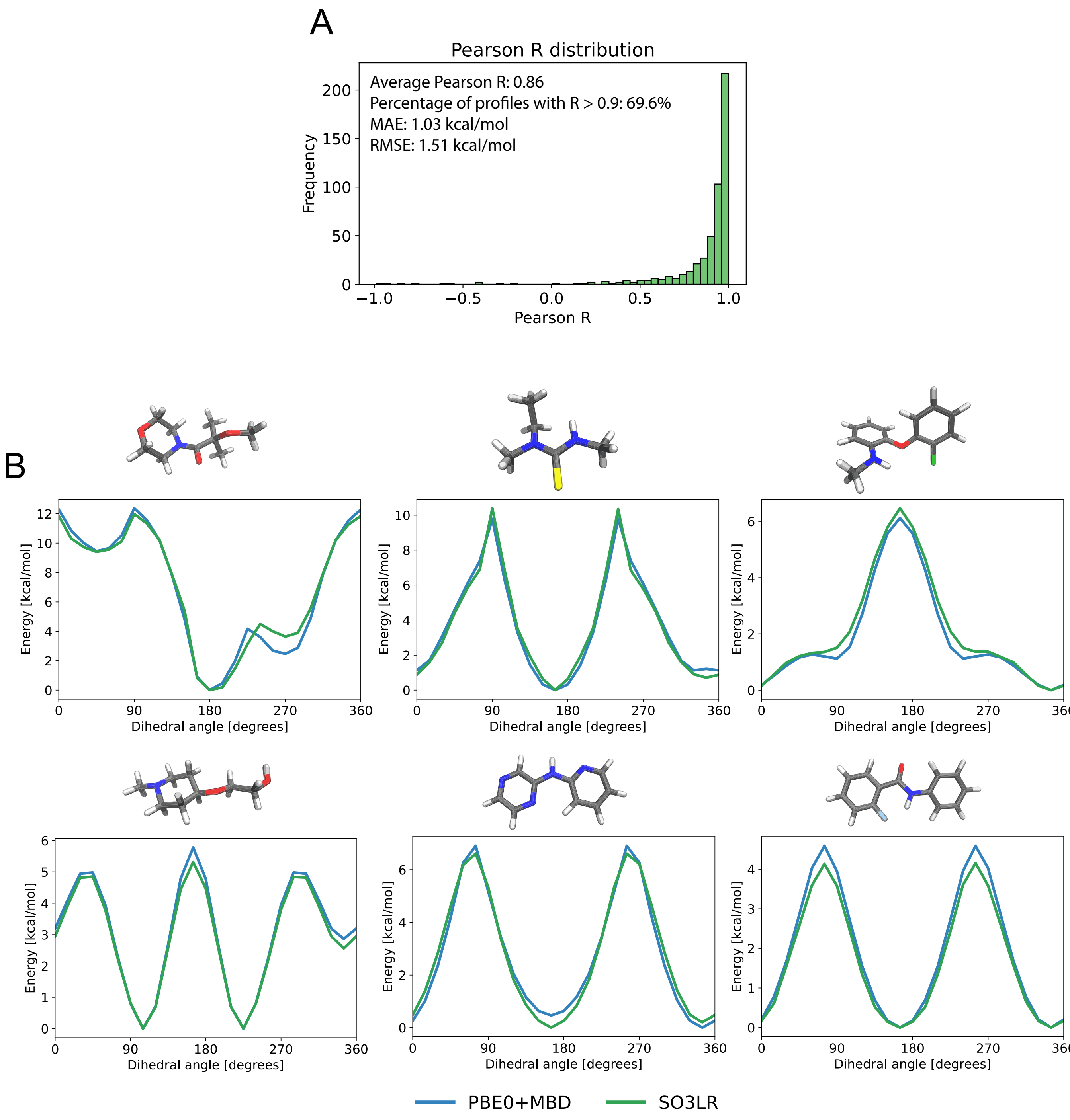

Fig. S3. TorsionNet500 Benchmark. Comparison of energies predicted by SO3LR with the TorsionNet500 benchmark [28], recomputed at the PBE0+MBD level of theory. **A** Histogram of Pearson R coefficients, with additional metrics shown in the inset. **B** Torsional profiles for six molecules. The absence of certain functional groups (e.g., triazole and trifluoromethylthio groups) in the training set leads to higher average errors. In contrast, torsional profiles commonly encountered in biosimulations are predicted accurately.

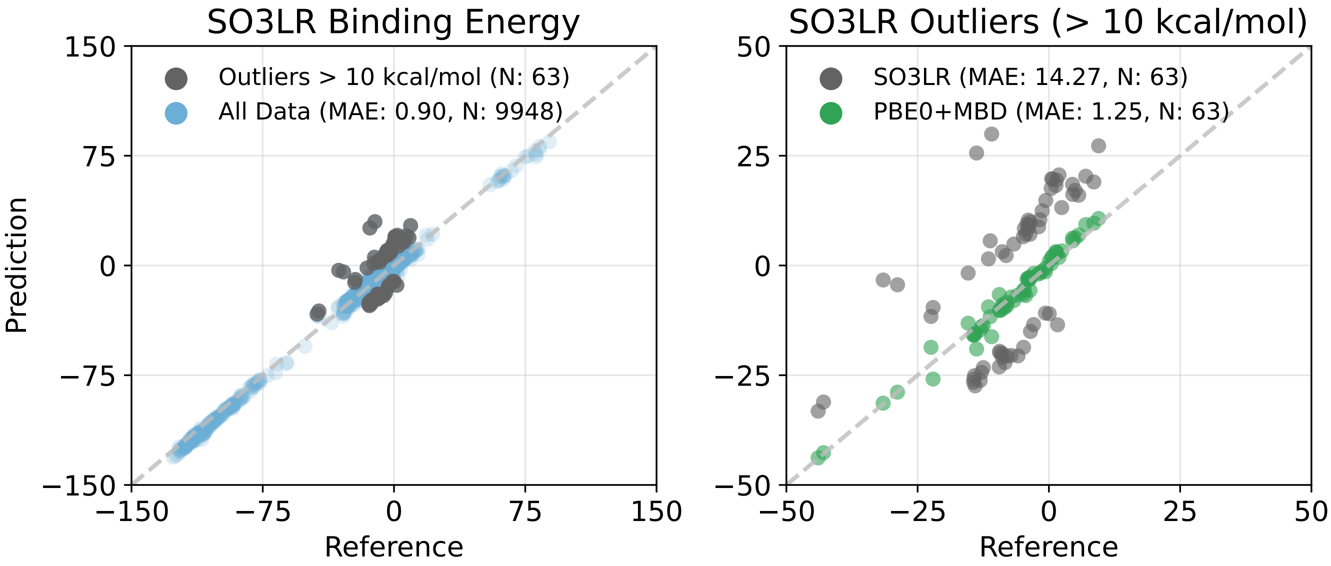

Fig. S4. SAPT10k outliers analysis. Outlier structures with binding energy errors >10 kcal/mol (left) include exotic molecules such as ClF, $P(CNO)_3$, and PH_2NO_2. Recalculation of the SO3LR outliers (63 dimers) at the PBE0+MBD/tight level (right) yields a mean absolute error (MAE) of 1.25 kcal/mol, with a maximum error of 5.33 kcal/mol. These results confirm that the errors arise from the absence of these motifs in the training set, rather than from limitations of the reference theory.

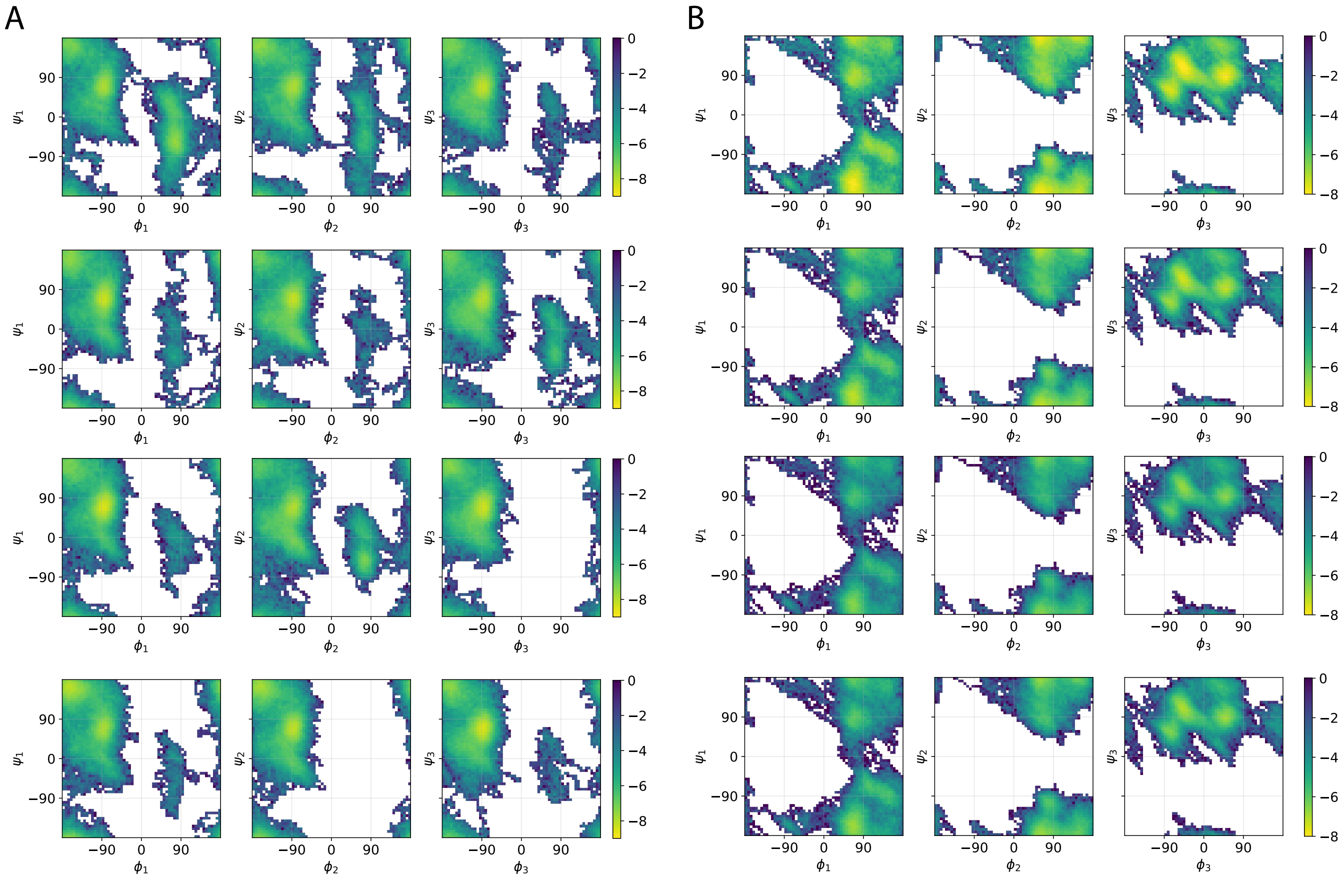

Fig. S5. Simulations of small biomolecular fragments. Ramachandran plots (ϕ/ψ dihedrals) for (A) $AcAla_3NMe$ and (B) stachyose molecules from the MD22 dataset. SO3LR simulations at 500 K for 500 ps. Trajectory is sampled every 1 fs. The Boltzmann-inverted scale is shown in kcal/mol.

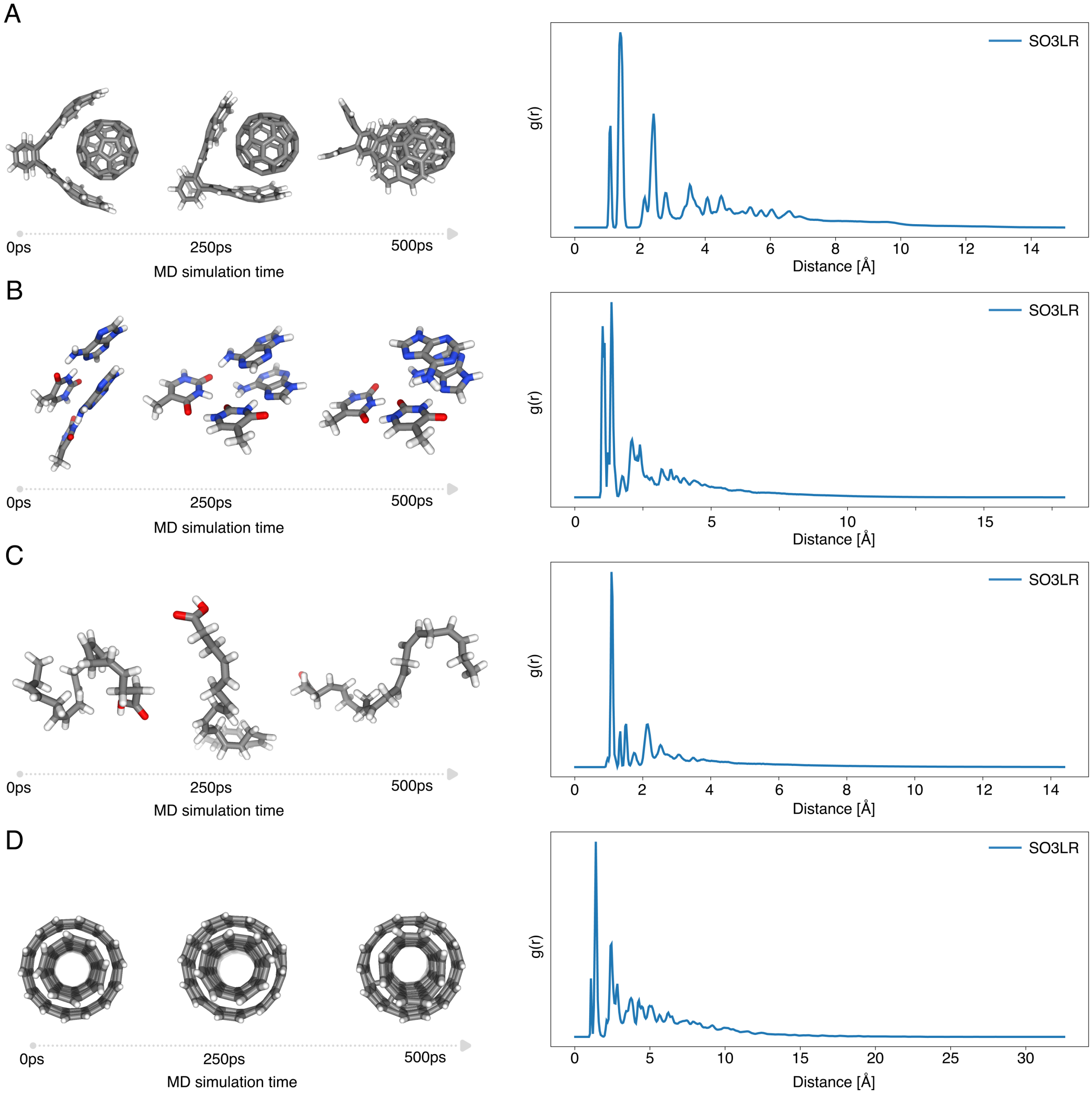

Fig. S6. Simulation of structures from the MD22 dataset. Snapshots of the simulation at 0 ps, 250 ps and 500 ps (left) and the corresponding radial distribution function $g(r)$ computed over frames sampled every 1 ps (right) for the **(A)** buckyball catcher, **(B)** AT-AT, **(C)** DHA, and **(D)** double-walled nanotube. Simulations were performed for 500 ps at 300 K.

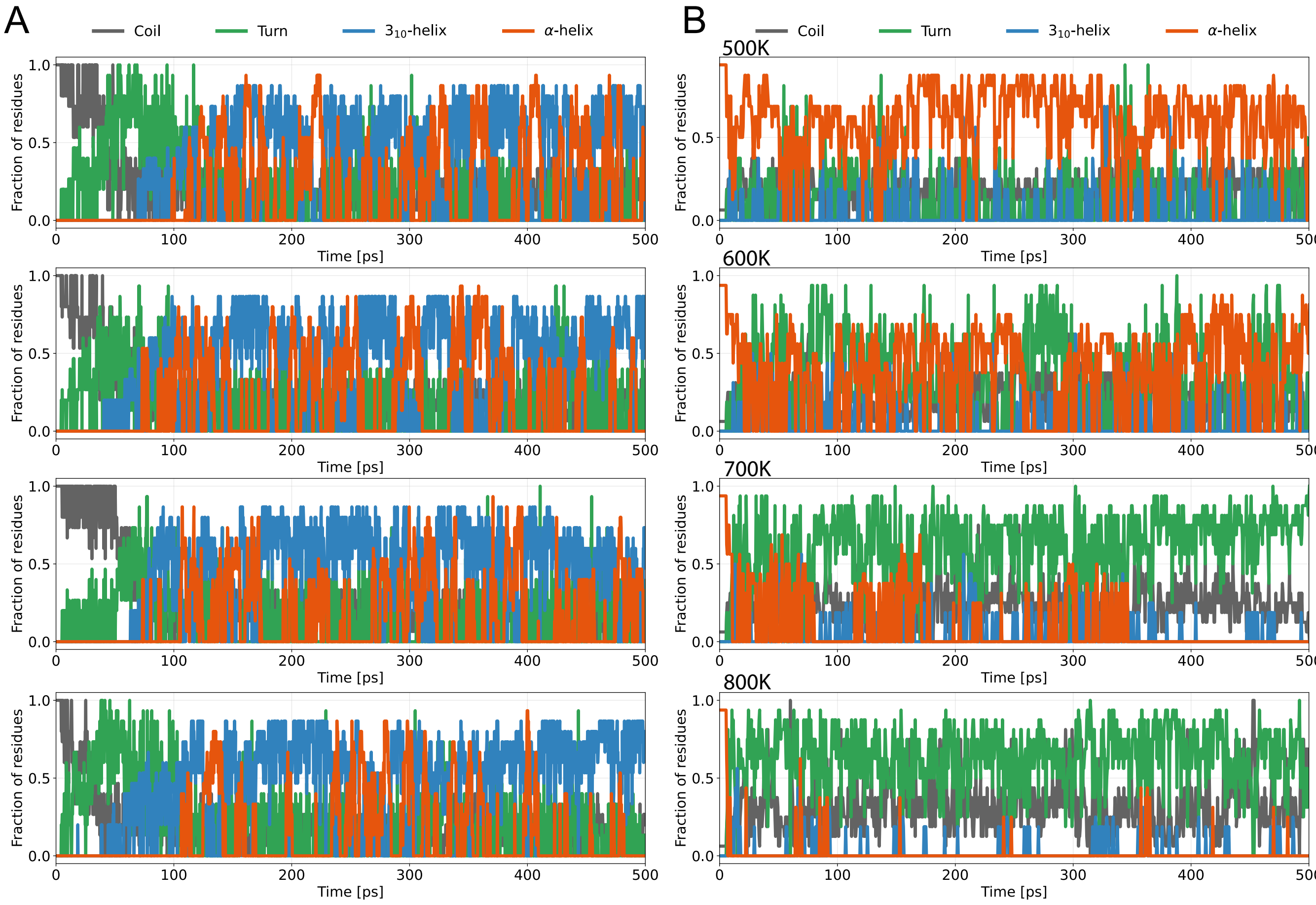

Fig. S7. Polyalanine simulation. Secondary structural motifs of (A) four folding trajectories of extended $AcAla_{15}NMe$ at 300 K in the gas phase and (B) four trajectories starting from the folded $AcAla_{15}LysH^{+}$ at 500, 600, 700, and 800 K. STRIDE was used for secondary structure assignment [19].

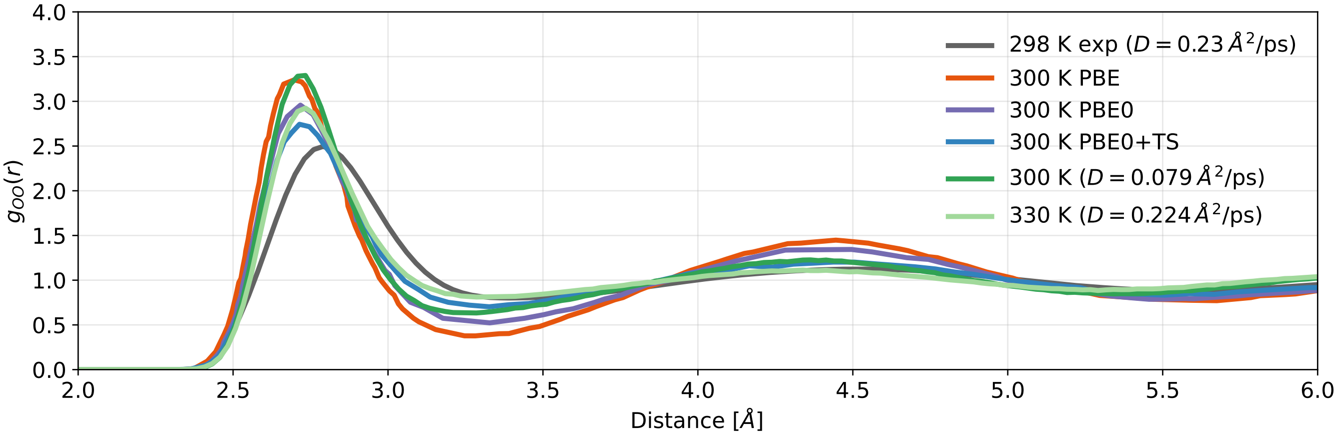

Fig. S8. Oxygen-oxygen radial distribution function for bulk water. The SO3LR values were calculated from NPT molecular dynamics simulations of 4096 water molecules, run for 500 ps, with observables averaged over the final 250 ps. DFT values (PBE, PBE0, PBE0+TS) were taken from Ref. 29. The diffusion coefficients of water at 300 and 330 K, obtained using the model with a 12 Å long-range cutoff, are specified in the legend. The experimental values were taken from Refs. 30, 31.

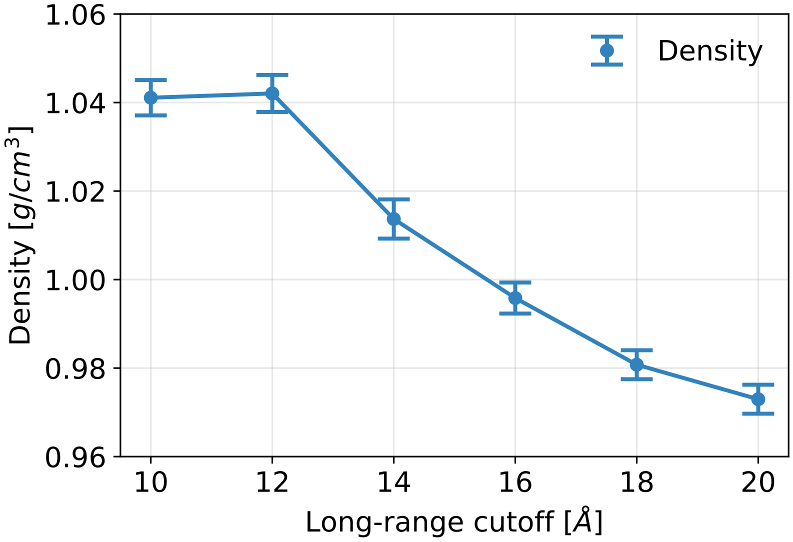

Fig. S9. Dependence of the water density on long-range cutoff at 300 K. We investigated the convergence of the density as a function of the cutoff for long-range interactions (see 'long-range cutoff' subsection). The water density varies between 1.04 and 0.97 g/cm^3 for long-range cutoffs of 10–20 Å.

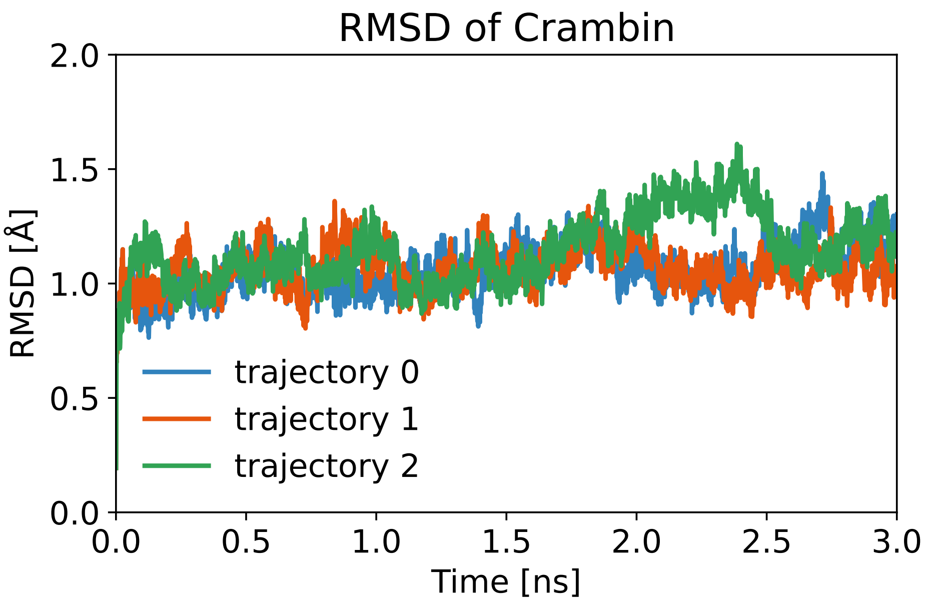

Fig. S10. Crambin RMSD. Root mean square deviations of three crambin trajectories simulated with SO3LR with respect to the initial frame.

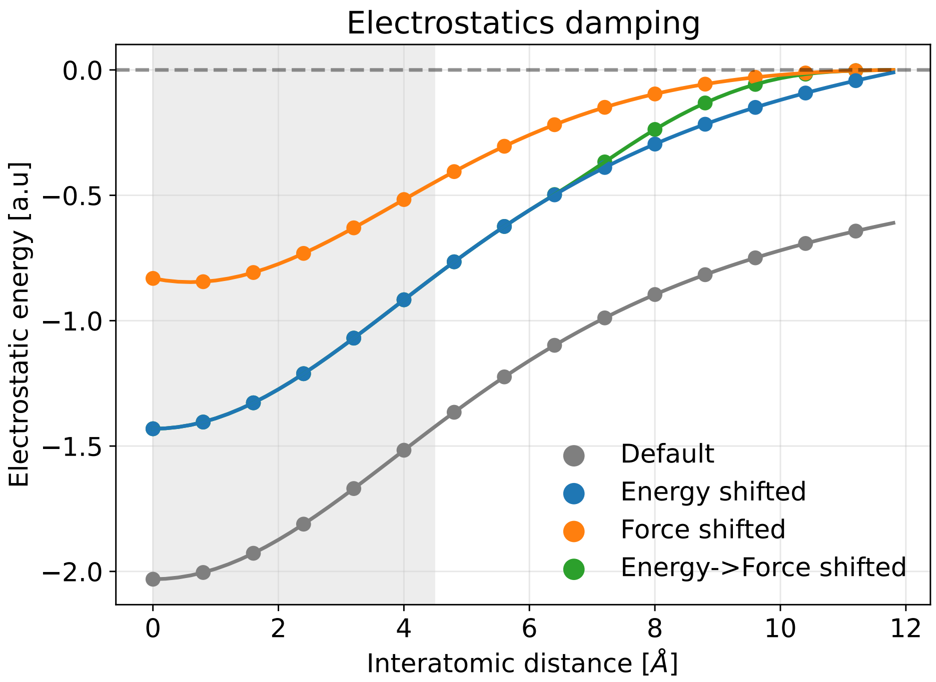

Fig. S11. Switching electrostatic interactions. The model was trained with a damped $\mathrm{erf}(r_{ij}/4)/r_{ij}$ electrostatic potential on gas-phase data, with a 100 Å long-range cutoff that recovers all neighbours and a 4.5 Å short-range cutoff. In simulations with periodic boundary conditions, we employ a long-range cutoff of 12 Å to balance accuracy and computational efficiency. The potential at short-range should be the same as the one the model was trained with to maintain the learned balance between different terms. Simultaneously, the potential should smoothly transition to zero at the long-range cutoff to ensure that the potential is the exact integral of the force and to avoid introducing discontinuities in the forces. Therefore, we smoothly switch between the energy-shifted (blue curve) and force-shifted (orange curve) potentials to obtain the final potential (green curve). Dispersion interactions are smoothly energy-shifted starting 2 Å before long-range cutoff.

REFERENCES

[1] V. Blum, R. Gehrke, F. Hanke, P. Havu, V. Havu, X. Ren, K. Reuter, and M. Scheffler, Ab initio molecular simulations with numeric atom-centered orbitals, Comput. Phys. Commun. **180**, 2175 (2009).

[2] X. Ren, P. Rinke, V. Blum, J. Wieferink, A. Tkatchenko, A. Sanfilippo, K. Reuter, and M. Scheffler, Resolution-of-identity approach to hartree–fock, hybrid density functionals, rpa, mp2 and gw with numeric atom-centered orbital basis functions, New J. Phys. **14**, 053020 (2012).

[3] O. T. Unke, S. Chmiela, M. Gastegger, K. T. Schütt, H. E. Sauceda, and K.-R. Müller, SpookyNet: Learning force fields with electronic degrees of freedom and nonlocal effects, Nat. Commun. **12**, 7273 (2021).

[4] T. Frank, O. Unke, and K.-R. Müller, So3krates: Equivariant attention for interactions on arbitrary length-scales in molecular systems, Adv. Neural Inf. Process. Syst. **35**, 29400 (2022).

[5] J. T. Frank, O. T. Unke, K.-R. Müller, and S. Chmiela, A euclidean transformer for fast and stable machine learned force fields, Nat. Commun. **15**, 6539 (2024).

[6] K. T. Schütt, H. E. Sauceda, P.-J. Kindermans, A. Tkatchenko, and K.-R. Müller, SchNet – a deep learning architecture for molecules and materials, J. Chem. Phys. **148**, 241722 (2018).

[7] O. T. Unke and M. Meuwly, PhysNet: A neural network for predicting energies, forces, dipole moments, and partial charges, J. Chem. Theory Comput. **15**, 3678 (2019).

[8] S. Batzner, A. Musaelian, L. Sun, M. Geiger, J. P. Mailoa, M. Kornbluth, N. Molinari, T. E. Smidt, and B. Kozinsky, E (3)-equivariant graph neural networks for data-efficient and accurate interatomic potentials, Nat. Commun. **13**, 2453 (2022).

[9] J. F. Ziegler, J. P. Biersack, and U. Littmark, The stopping and range of ions in solids (Pergamon Press, New York, 1985).

[10] C. J. Fennell and J. D. Gezelter, Is the ewald summation still necessary? pairwise alternatives to the accepted standard for long-range electrostatics, J. Chem. Phys. **124**, 234104 (2006).

[11] D. Wolf, P. Keblinski, S. Phillpot, and J. Eggebrecht, Exact method for the simulation of coulombic systems by spherically truncated, pairwise r- 1 summation, J. Chem. Phys. **110**, 8254 (1999).

[12] S. J. Reddi, S. Kale, and S. Kumar, On the convergence of adam and beyond, arXiv preprint arXiv:1904.09237 10.48550/arXiv.1904.09237 (2019).

[13] J. L. Ba, J. R. Kiros, and G. E. Hinton, Layer normalization, arXiv preprint arXiv:1607.06450 10.48550/arXiv.1607.06450 (2016).

[14] S. Schoenholz and E. D. Cubuk, Jax md: a framework for differentiable physics, Adv. Neural Inf. Process. Syst. **33**, 11428 (2020).

[15] S. Nosé, A unified formulation of the constant temperature molecular dynamics methods, J. Chem. Phys **81**, 511 (1984).

[16] W. G. Hoover, Canonical dynamics: Equilibrium phase-space distributions, Phys. Rev. A **31**, 1695 (1985).

[17] G. J. Martyna, M. L. Klein, and M. Tuckerman, Nosé–hoover chains: The canonical ensemble via continuous dynamics, J. Chem. Phys **97**, 2635 (1992).

[18] E. Bitzek, P. Koskinen, F. Gähler, M. Moseler, and P. Gumbsch, Structural relaxation made simple, Phys. Rev. Lett. **97**, 170201 (2006).

[19] D. Frishman and P. Argos, Knowledge-based protein secondary structure assignment, Proteins Struct. Funct. Bioinform. **23**, 566 (1995).

[20] G. Pranami and M. H. Lamm, Estimating error in diffusion coefficients derived from molecular dynamics simulations, J. Chem. Theory Comput. **11**, 4586 (2015).

[21] E. J. Maginn, R. A. Messerly, D. J. Carlson, D. R. Roe, and J. R. Elliot, Best practices for computing transport properties 1. self-diffusivity and viscosity from equilibrium molecular dynamics [article v1. 0], Living J. Comp. Mol. Sci. **1**, 6324 (2019).

[22] D. Bang, V. Tereshko, A. A. Kossiakoff, and S. B. H. Kent, Role of a salt bridge in the model protein crambin explored by chemical protein synthesis: X-ray structure of a unique protein analogue, [v15a]crambin-α-carboxamide, Mol. BioSyst. **5**, 750 (2009).

[23] K. T. Schütt, P. Kessel, M. Gastegger, K. A. Nicoli, A. Tkatchenko, and K.-R. Müller, SchNetPack: A Deep Learning Toolbox For Atomistic Systems, J. Chem. Theory Comput. **15**, 448 (2019).

[24] K. T. Schütt, S. S. P. Hessmann, N. W. A. Gebauer, J. Lederer, and M. Gastegger, SchNetPack 2.0: A neural network toolbox for atomistic machine learning, J. Chem. Phys. **158**, 144801 (2023).

[25] A. Mølgaard and S. Larsen, A branched N-linked glycan at atomic resolution in the 1.12Å structure of rhamnogalacturonan acetylesterase, Acta Crystallogr. D **58**, 111 (2002).

[26] C. J. Dickson, R. C. Walker, and I. R. Gould, Lipid21: complex lipid membrane simulations with amber, J. Chem. Theory Comput. **18**, 1726 (2022).

[27] D. R. Roe and T. E. Cheatham III, Ptraj and cpptraj: software for processing and analysis of molecular dynamics trajectory data, J. Chem. Theory Comput. **9**, 3084 (2013).

[28] B. K. Rai, V. Sresht, Q. Yang, R. Unwalla, M. Tu, A. M. Mathiowetz, and G. A. Bakken, Torsionnet: A deep neural network to rapidly predict small-molecule torsional energy profiles with the accuracy of quantum mechanics, J. Chem. Inf. Model. **62**, 785 (2022).

[29] R. A. DiStasio, B. Santra, Z. Li, X. Wu, and R. Car, The individual and collective effects of exact exchange and dispersion interactions on the ab initio structure of liquid water, J. Chem. Phys. **141**, 084502 (2014).

[30] A. K. Soper, The radial distribution functions of water as derived from radiation total scattering experiments: is there anything we can say for sure?, Int. Sch. Res. Notices **2013**, 279463 (2013).

[31] R. Mills, Self-diffusion in normal and heavy water in the range 1-45. deg., J. Phys. Chem. **77**, 685 (1973).
